# Proceedings of the Fourteenth International Society of Sports Nutrition (ISSN) Conference and Expo: Phoenix, AZ, USA. 22-24 June 2017

**DOI:** 10.1186/s12970-017-0188-5

**Published:** 2017-09-12

## P1 Effects of a short-term paleolithic diet on fitness and body composition

### Michael Dahlinghaus (dahinghausmichaelf@sau.edu)

#### St. Ambrose University, Davenport IA, USA

**Background**

A paleolithic diet aims at mimicking the type of foods that hunter-gatherers would have most likely consumed. While the adoption and popularity of a paleolithic diet has raised among the general population and fitness communities, there has been little research documenting its effect on general fitness (max strength, cardiorespiratory endurance, muscular endurance) and body composition. The purpose of this randomized controlled study was to study the effects of adopting a paleolithic diet (PD) on general fitness performance and body composition in an active population.

**Methods**

Nineteen (mean ± SD age = 20.89 ± 2.07) physically active males (n = 13) males and females (n = 6) volunteered to participate in this study. Subjects were randomly divided into two groups: a paleolithic group (PG) (males, n = 8; females, n = 3) and control group (males, n = 5; females, n = 3). Both groups underwent a pre fitness and body composition assessment. This assessment included height, weight, blood pressure (BP), body fat percentage, one repetition maximum (1RM) back squat, 1RM bench press, push up test to failure (PUT), and a Vo2 max test (1.5 mile run). The PG was then instructed to follow a PD for three weeks. This eliminated grains, dairy, and processed food from their normal diet. The control group maintained their normal diet. Both groups were instructed to continue their normal exercise program (exercising ≥3x a week). After three weeks, both groups underwent a post-test consisting of the same initial assessments. A paired sample t-test was used to investigate statistically significant differences between the pre and post-tests for the experimental and control group.

**Results**

For the experimental group, mean weight decreased significantly by 3.27lbs (P < .05) and systolic BP by 7.46 (P < .043). Mean PUT increased by 7.36 (P < .001). The only significant variable in the control group was a mean increase in PUT by 3 (P < .03).

**Conclusion**

This short intervention did find some favorable effect consuming a Paleolithic Diet. A PD may be an effective means at reducing weight, decreasing systolic blood pressure, while maintaining physical fitness in active populations.

## P2 Comparison of micronutrient intakes between flexible dieting and strict dieting male bodybuilders

### Ahmed Ismaeel (Ahmed_Ismaeel@baylor.edu)

#### Nutrition Sciences, Baylor University, Waco, TX, 76706, USA

**Background**

A recent literature review [1] identified that many previous older studies have reported deficiencies in intakes of several micronutrients in bodybuilders Notably, a new “flexible” dieting strategy, popularly known as “if it fits your macros” or “IIFYM,” has recently become widespread. In contrast to restriction-based dieting, IIFYM instead focuses on monitoring individual macronutrient intake, with less regard for the specific foods consumed. The purpose of this study was to assess selected micronutrient intakes of male competitive bodybuilders and to compare the intakes of those who follow flexible dieting and strict dieting regimens.

**Methods**

Subjects for this study were male competitive bodybuilders, recruited online via social media outlets. The Diet History Questionnaire, Version 2.0 (DHQ-II) [2], a semi-quantitative food frequency questionnaire (FFQ), was used for this study to assess micronutrient intakes. A pooled *t*-test was used to test whether flexible dieters and strict dieters consume, on average, the same mean amount of each micronutrient, with α being significant at p < 0.05.

**Results**

Thirty individuals were included in the final analyses, of which 14 followed flexible dieting and 16 followed strict dieting. Ages ranged from 20 to 50 years old, with a mean age of 29.1 years (SD = 6.9). Subjects consumed an average of 2577.2 (SD = 955.1) kilocalories, with an average fat intake of 83.6 g (SD = 41.3), an average carbohydrate intake of 324.4 g (SD = 105.2), and an average protein intake of 163.4 (SD = 70.4). Of the vitamins and minerals assessed in this study, vitamin A, vitamin D, vitamin E, and potassium were consumed at levels below the RDA. In addition, dietary fiber intake was below current recommendations. Nutrient data for all subjects are summarized in Table 1.

There were no significant differences between male flexible and strict dieting bodybuilders when mean nutrient intakes were compared. However, in the strict dieting group, average intakes were higher for all nutrients, and a greater proportion of individuals met the RDA/AI. Table 2 highlights the differences in nutrient consumption between the two groups.

**Conclusions**

Based on the results of this study, male competitive bodybuilders may need to pay more attention to their intakes of certain micronutrients. Vitamins A, D, and E, and dietary fiber were all consumed below recommended amounts. Vitamins A and E are both important antioxidants, vitamin D has been associated with muscle strength [3], and dietary fiber can increase satiety, regulate blood glucose, and is inversely associated with body fat [4]. While there were no large differences between the nutrient intakes of males who follow flexible dieting compared to strict dieting, the greater proportion of individuals in the flexible dieting group who were not meeting the RDA for several nutrients suggests that this group should especially take their micronutrition into greater consideration.

**References**

1. Helms ER, Aragon AA, Fitschen P: Evidence-based recommendations for natural bodybuilding contest preparation: nutrition and supplementation. *J Int Soc Sports Nutr.* 2014, 11:20.

2. Diet History Questionnaire, Version 2.0. National Insitutes of Health, Epidemiology and Genomics Research Program, National Cancer Institute. 2010.

3. Chiang CM, Ismaeel A, Griffis RB, Weems S: Effects of vitamin D supplementation on muscle strength in athletes a systematic review. *J Strength Cond Res.* 2017, 31(2): 566-574.

4. Slavin JL: Dietary fiber and body weight. *Nutrition*. 2005, 21(3): 411-418.

**Table 1 (abstract P2).** Dietary intake in male competitive bodybuilders

Nutrient	Reported Intake	RDA/AI	% RDA/AI met
**Fiber**	**26.9 (12.9) g**	**38 g**	**70.8%**
**Vitamin A**	**776.4 (418.3) mcg**	**900 mcg**	**86.3%**
**Vitamin D**	**372.3 (574.0) IU**	**600 IU**	**62.05%**
**Vitamin E**	**13.9 (8.3) mg**	**15 mg**	**92.7%**
Vitamin C	165.7 (118.8) mg	90 mg	184.1%
Thiamin	1.4 (1.0) mg	1.2 mg	200.0%
Riboflavin	4.0 (1.3) mg	1.3 mg	307.7%
Niacin	51.7 (28.5) mg	16 mg	323.1%
Pyridoxine	4.2 (1.9) mg	1.3 mg	323.1%
Folic acid	774.4 (401.5) mcg	400 mcg	193.6%
Cobalamin	9.6 (5.3) mcg	2.4 mcg	400.0%
Calcium	1609.4 (523.5) mg	1000 mg	160.9%
Phosphorous	2129.0 (915.2) mg	700 mg	304.1%
Magnesium	591.9 (770.9) mg	420 mg	140.9%
Iron	26.5 (13.0) mg	8 mg	331.2%
Zinc	19.0 (8.8) mg	11 mg	172.7%
Sodium	4670.7 (2232.8) mg	1500 mg	311.4%
**Potassium**	**4413.2 (1774.4) mg**	**4700 mg**	**93.9%**

Note: Data presented as mean (SD) when applicable, bold font indicates nutrients below RDA/AI

**Table 2 (abstract P2).** Comparison of intakes between flexible and strict dieting males

Nutrient	Flexible Dieters (Fl)	Strict Dieters (Str)	*P*-value	Proportion Below RDA/AI
Calories	2490.2 (899.2)	2644.9 (1045.0)	0.38	N/A
Fat	84.0 (34.2) g	84.3 (42.3) g	0.51	N/A
Carbohydrate	316.9 (101.6) g	330.1 (113.7) g	0.41	N/A
Protein	161.4 (29.6) g	169.1 (66.9) g	0.21	N/A
Fiber	25.5 (17.9) g	28.0 (8.2) g	0.36	Fl: 9/14 Str: 10/16
Vitamin A	771.3 (434.9) mcg	780.4 (431.5) mcg	0.48	Fl: 8/14 Str: 7/16
Vitamin D	304.5 (607.4) IU	388.2 (516.5) IU	0.14	Fl: 10/14 Str: 12/16
Vitamin E	11.9 (9.1) mg	15.4 (7.8) mg	0.21	Fl: 10/14 Str: 7/16
Vitamin C	156.2 (146.2) mg	173.0 (101.4) mg	0.40	Fl: 7/14 Str: 1/16
Thiamin	2.3 (1.1) mg	2.4 (0.9) mg	0.45	Fl: 1/14 Str: 0/16
Riboflavin	3.9 (1.6) mg	4.0 (1.2) mg	0.46	Fl: 0/14 Str: 0/16
Niacin	46.8 (21.3) mg	55.6 (33.9) mg	0.28	Fl: 0/14 Str: 0/16
Pyridoxine	4.1 (2.1) mg	4.2 (1.9) mg	0.46	Fl: 0/14 Str: 0/16
Folic acid	705.1 (515.6) mcg	828.2 (309.0) mcg	0.28	Fl: 6/14 Str: 2/16
Cobalamin	9.2 (4.3) mcg	9.9 (6.2) mcg	0.40	Fl: 0/14 Str: 1/16
Calcium	1548.8 (670.1) mg	1656.6 (413.9) mg	0.35	Fl: 4/14 Str: 0/16
Phosphorous	2020.4 (932.2) mg	2213.4 (949.0) mg	0.35	Fl: 0/14 Str: 0/16
Magnesium	515.6 (281.5) mg	651.2 (262.9) mg	0.17	Fl: 7/14 Str: 3/16
Iron	24.1 (14.5) mg	28.3 (12.3) mg	0.27	Fl: 0/14 Str: 0/16
Zinc	18.4 (9.4) mg	19.5 (8.8) mg	0.41	Fl: 1/14 Str: 0/16
Sodium	4488.7 (2077.8) mg	4812.2 (2461.3) mg	0.39	Fl: 0/14 Str: 0/16
Potassium	4095.5 (2203.4) mg	4660.4 (1451.0) mg	0.27	Fl: 10/14 Str: 5/16

Note: Data presented as mean (SD) when applicable, *P*-value is for pooled *t*-test, testing H_0_: *μ*_2_ = *μ*_1_ against H_a_: *μ*_2_ > *μ*_1_, proportion below RDA/AI refers to the number of individuals in each population not meeting RDA/AI for each nutrient out of total population

## P3 Evaluation of the effects of two doses of alpha glycerylphosphorylcholine on thyroid stimulating hormone levels

### David Bellar^1^, Jason Soileau^2^, Lena Marcus ^1^, Lawrence W. Judge^3^

#### ^1^School of Kinesiology, University of Louisiana at Lafayette, Lafayette LA USA; ^2^Pennington Biomedical Research Center, Louisiana State University, Baton Rouge LA USA; ^3^School of Kinesiology, Ball State University, Muncie IN USA

##### **Correspondence:** David Bellar (dbellar@louisiana.edu)

**Background**

Recent studies have suggested that alpha glycerylphosphorylcholine (A-GPC) may be an effective ergogenic aid that can provide cognitive benefit. A-GPC has been shown to enhance acetylcholine levels and can cross the blood brain barrier. Increasing CNS acetylcholine can affect dopamine levels, which can in turn affect other hormones such as Thyroid stimulating hormone (TSH). The present study was designed to assess the efficacy of two doses of A-GPC in comparison to placebo for decreasing thyroid stimulating hormone, a signal of increased dopamine.

**Methods**

Forty-eight healthy, college aged males volunteered for the present study and underwent baseline assessment of fitness. Following this assessment participants were randomly assigned to groups consisting of 500 mg A-GPC, 250 mg A-GPC, or Placebo who reported back to the lab in the morning hours (0700) in a fasted state. Blood samples were collected 1 hour and 2 hours post dosing. Serum free choline and thyroid stimulating hormone were analyzed via commercially available ELISA assays.

**Results**

Serum free choline was found to be elevated in the two A-GPC groups as compared to placebo (132% and 59% respectively). Serum TSH was found to be significantly depressed in the 500 mg A-GPC group compared to other treatments (p < 0.04).

**Conclusions**

Based upon this evidence, and previous evidence regarding A-GPC, more study should be devoted to the potential neurological benefits associated with this supplement.

**Acknowledgements**

This study was funded in part by a research grant from Chemi Nutra, Austin Tx.

## P4 The effect of detraining and body composition on anaerobic power production in female collegiate athletes

### Natalie H. Fry, Troy M. Purdom, Kyle S. Levers, Lindsey Brown, Deanna E. Wetzel, Jake R. Giles

#### Department of Health, Athletic Training, Recreation, and Kinesiology, Longwood University, Farmville, VA, USA

##### **Correspondence:** Troy M. Purdom (purdomtm@longwood.edu)

**Background**

It is well known that having a favorable body composition (less fat mass, more fat free mass) is ideal for sports performance applications. Fat mass does not contribute to force/power development and can impact performance negatively [1]. Body composition changes may occur as a result of short-term detraining, and therefore may alter anaerobic performance [2]. Therefore, the purpose of this study was to investigate the effect of nine weeks of detraining on body composition and anaerobic performance.

**Methods**

Sixteen female collegiate soccer athletes (Mean ± SD: 19.3 ± 1.08 yrs; 62.17 ± 5.95 kg; 164.81 ± 5.9 cm; 21.46 ± 3.42%BF; 107.43 ± 9.5FFM(kg)) were assessed at two separate time blocks: after the competitive season and again after nine weeks of detraining. Each testing block included two testing days separated by 24 hours. Each subject arrived having refrained from food for four hours, caffeine for 12 hours and alcohol and exercise for 24 hours. Informed consent and demographic information were collected in addition to body composition using a two-component model (three-site skinfold analysis) prior to physical testing. Day one for each testing block consisted of a counter movement vertical jump (CMJ) and day two consisted of the 40-yard dash and the running anaerobic sprint test (RAST). Paired t-tests were used to evaluate statistical differences between testing blocks. Performance measures are reported in absolute values as watts (W) and relative power in watts per kg (W/kg). All values are expressed as mean ± SD.

**Results**

Statistical analysis revealed that nine weeks of detraining induced a significant decrease in both peak vertical power and average horizontal power. A 4.44% decrease in CMJ was reported (*p* = 0.003; pre 48.82 ± 5.73, post 46.59 ± 5.54 W/kg) and average horizontal RAST power decreased by 6.13% (*p* = 0.031; pre 343.26 ± 57.25, post 310.93 ± 28.68 W), respectively. No significant changes in %BF (*p* = 0.40) or FFM (kg) (*p* = 0.31) were observed. Peak horizontal power (40-yard dash) was not significantly different between testing blocks (*p* > 0.05).

**Conclusions**

While FFM did not change throughout the detraining period, peak vertical and average horizontal power decreased agreeing with previous literature [2]. This suggests that cellular changes are responsible for the reduction in power, not body composition. However, the lack of change in peak horizontal power warrants further inquiry. The results show that detraining may reduce anaerobic power despite the lack of changes in body composition.

**References**

1. Buskirk, ER. Mendez, J. Sports science and body composition analysis: emphasis on cell and muscle mass. *Medicine Science in Sports and Exercise*. 1984, 16(6): 584-595

2. Winters, KM. Snow, CM. Detraining reverses positive effects of exercise on the musculoskeletal system in premenopausal women. *Journal of Bone and Mineral Research*. 2000, 15: 2495-2503.

## P5 The effects of a pre-workout supplement on strength, endurance and mood

### Cassandra Carson, Anya Ellerbroek, Leo Vargas, Corey Peacock, Tobin Silver, Jose Antonio

#### Department of Health and Human Performance, Nova Southeastern University, Davie FL, USA

##### **Correspondence:** Jose Antonio (ja839@nova.edu)

**Background**

The popularity of pre workout supplements to help increase performance has increased in both recreational as well as professional athletes in the past few years. It is unclear whether caffeine alone or a combination of caffeine with other ingredients do indeed have an ergogenic effect. Therefore, the purpose of this study was to assess the acute effects of consuming a pre-workout supplement on indices of muscular strength, endurance and mood states. A randomized, double-blind, placebo-controlled crossover design was utilized in this investigation.

**Methods**

Fourteen moderate to highly-trained recreational athletes (7 female, 7 male) participated in this investigation (Table 3). Their body composition was assessed via the DEXA (Hologic Model Horizon W).

Subjects came to the lab twice with at least 7 days between testing sessions. The consumption of product or placebo was randomized. They arrived at the lab 3 hours fasted with no prior exercise that day. Subsequently, they consumed the supplement or placebo (mixed with 8-12 ounces of water) 30 minutes prior to testing. The pre-workout supplement (Athelite Nutrition Inc.) contained 15.62 grams per serving, 25 kcals, that consisted of a proprietary blend including caffeine (as green coffee bean extract), L-theanine, black pepper extract, micronized creatine monohydrate, CarnoSyn® beta-alanine, Huperzine A, N-Acetyl L-carnitine, Nitrosigine®), or placebo. The placebo was a similar tasting drink with an equal amount of caffeine. Participants’ mood was also assessed via a profile mood states questionnaire (POMS) 30 minutes after product or placebo was consumed. After taking the POMS questionnaire, subjects had their exercise performance assessed via the 1-RM bench press followed by bench press repetitions to failure at 60% of 1-RM with 30 seconds rest between sets (3 total sets).

**Results**

There were significant differences (p < 0.05) between the supplement and placebo for the number of repetitions to failure as well as total weight lifted (Table 4). However, there were no differences for any of the other parameters measured (Table 5).

**Conclusion**

The results demonstrated that the acute consumption of a pre-workout supplement can enhance muscular endurance; however, it has no effect on strength or mood states.

**Acknowledgments**

This study was unfunded. However, product and placebo was provided by Athelite Nutrition.

**Table 3 (abstract P5).** Physical Characteristics

Age yr	33.1±5.7
Height cm	171.1±9.1
Weight kg	78.8±21.9
BMC kg	2.8±0.5
Fat Mass kg	18.7±8.3
Lean Mass kg	57.4±17.2
% Body Fat	23.9±7.0

Data are expressed as the mean±SD. Legend: BMC, bone mineral content, cm – centimeters, kg – kilograms, yr – years. n=14 (7 female, 7 male)

**Table 4 (abstract P5).** Exercise Performance

	Placebo	Treatment
1-RM kg	94.0±52.2	92.5±52.3
Repetitions to Failure	19.0±4.7	22.7±4.0*
Total Weight Lifted^+^	1124.2±739.3	1290.4±818.3*

Data are expressed as the mean±SD. Legend: kg – kilograms. *treatment significantly different than placebo, p<0.05. ^+^total weight lifted was calculated by the number of repetitions times the weight lifted

**Table 5 (abstract P5).** Profile of Mood States

	Placebo	Treatment
Anger	3.3±5.1	1.6±2.8
Confusion	3.4±2.3	3.5±2.3
Depression	1.4±2.7	1.3±2.9
Fatigue	3.4±4.7	2.1±2.1
Tension	6.3±4.5	7.4±6.0
Vigor	17.6±6.9	16.9±7.4

Data are expressed as the mean±SD. There were no significant differences between groups

## P6 L-Isoleucine and L-leucine, may increase glucose uptake through insulin independent effects in healthy, inactive adults

### Daniel E. Newmire^1^, Eric Rivas^2^, Sarah E. Deemer^3^, and Victor Ben-Ezra^3^

#### ^1^University of Nebraska Omaha; Department Health, Physical Education, and Recreation; Omaha, NE; USA; ^2^The University of Texas Medical Branch; Department of Surgery; Galveston, TX; USA; ^3^Texas Woman’s University; Department of Kinesiology; Denton, TX; USA

##### **Correspondence:** Daniel E. Newmire (dnewmire@unomaha.edu)

**Background**

The co-ingestion BCAA’s with a carbohydrate drink has shown to synergistically promote hyperinsulinemia and reduce blood glucose. Particularly, L-Leucine (LEU) directly and indirectly facilitates pancreatic insulin (INS) secretion through gut hormone (incretin) responses; however, L-Isoleucine (ISO) has not shown the same result. Though not fully understood, amino acids may independently stimulate release of gut (incretin) hormones glucagon-like peptide-1 (GLP-1) and glucose-dependent insulinotropic peptide (GIP). GLP-1 and GIP are suggested to be 50-70% responsible for regulating insulin secretion after oral glucose ingestion. However, it is unknown how LEU and ISO independently or synergistically stimulate incretin hormones. Therefore, the objective of the study was to determine the independent and combined effects of ISO and LEU on incretin (GLP-1, GIP) responses and subsequently their associations with glucose (GLU), C-peptide (CP), INS, and glucagon (GCG) concentrations in healthy, inactive adults.

**Methods**

12-healthy, inactive adults (Mean ± SEM; 6 M/F; age 27.4 ± 2.0 y; lean body mass (LBM) 48.6 ± 4.67 kg; body fat 34.1 ± 2.96%) completed 4-trials in a randomized, single-blinded fashion: A standardized ingestion to 0.3 g·kg^-1^·LBM^-1^; 12-17 g; 1) ISO; 2) LEU; 3) equal combination of ISO + LEU; and 4) placebo (PLA, 3.5 g inert stevia). Venous samples were taken at baseline, 6, 10, and 30 min for the analysis of dependent variables GIP_Total_, GLP-1_Active_, CP, INS, and GCG (quantified via MAGPIX) and GLU (YSI 2900). A 2-way (treatment x time) RMANOVA compared the incremental change (∆) and where appropriate, a Pearson’s r to express relationships for GLU, GIP_Total_, GLP-1_Active_, CP, INS, and GCG.

**Results**

Analysis (Mean ± SEM) revealed that each treatment reduced GLU by ∆ 3.8 mg/dl (main effect for treatment; *p <* 0.001) compared to PLA. INS and CP were not affected by any treatment. ISO + LEU, ISO, LEU increased GCG compared to PLA condition by ∆ 9 ng/l at 30 min (time x treatment interaction; *p* = 0.04). The incretin GLP-1_Active_ was unaffected by any treatment; however, ISO compared to LEU and PLA treatment increased peak GIP_Total_ by ∆ 3 pmol/l at 30 min (*p* = > 0.0002). No association was found between GIP_Total_ and INS during ISO treatment (*r* = 0.27, *r* ^*2*^ = 0.07, *p* = 0.42).

**Conclusion**

Overall, ISO and LEU induced a slight glucose reduction concurrent with a counter regulatory rise in GCG. Because we found no treatment effect by these amino acids on CP, INS, and GLP-1_Active_ concentrations, this data indicates that ISO and LEU may induce glucose uptake independent of insulin and GLP-1_Active_ responses. Additionally, no relationship was found between ISO induced GIP_Total_ and INS. This data support the concept that BCAA’s ISO and LEU may influence glucose uptake independent of glycemic hormones; however, this suggestion requires more rigorous investigation.

**Acknowledgement**

Participant compensation and supplemental treatments fabricated by Dymatize.

## P7 A randomized crossover, double-blinded, placebo-controlled study of the effects of acute oral ingestion of Bang® Pre-Workout Master Blaster™ on exercise performance and clinical safety markers

### Neil A. Schwarz^1^, Sarah K. McKinley-Barnard^1^, and Albert W. Pearsall^2^

#### ^1^Department of Health, Kinesiology, and Sport, University of South Alabama, Mobile, AL, USA; ^2^Department of Orthopaedic Surgery, University of South Alabama, Mobile, AL, USA

##### **Correspondence:** Neil A. Schwarz (neilschwarz@southalabama.edu)

**Background**

The purpose of this placebo-controlled, double-blind, crossover study was to determine the effects of acute oral ingestion of nutritional supplements containing either placebo or Bang® Pre-Workout Master Blaster^TM^ on exercise performance, serum hormone responses, and clinical safety markers in males.

**Methods**

Ten resistance-trained males participated in two exercise testing sessions consisting of the vertical jump (VJ), seated medicine ball throw (SMBT), and local muscular endurance tests for the bench press (BP) and leg extension (LE) exercises at 70% of one-repetition maximum. Participants consumed placebo (Fibersol-2) or Bang® approximately 30 minutes prior to performing each exercise session. Venous blood samples were obtained at baseline (BL), 30 minutes post-supplement (30PS), and 30 minutes post-exercise (30PX). Data were analyzed with paired t-tests or separate 2x3 (trial x time) within-within ANOVA (p < 0.05).

**Results**

No significant difference between trials was observed for SMBT distance or BP repetitions. Vertical jump (p < 0.01) and LE repetitions (p < 0.05) were significantly greater for the Bang® trial compared with placebo. A significant main effect for time was observed for serum cortisol (p = 0.02) and serum human growth hormone (HGH; p = 0.011) with no significant effect for trial or between trial and time. Serum cortisol was significantly decreased at 30PS compared with BL (p = 0.003). Serum HGH was significantly greater at 30PX compared with BL (p = 0.009) and 30PS (p = 0.014). A trend for HGH to be higher at 30PX for the Bang® trial compared with the placebo trial (p = 0.10) was noted. A statistically significant interaction between trial and time was observed for serum insulin-like growth factor-1 (IGF-1; p = 0.044). There was a significant effect of time for the Bang® trial (p = .010). Serum IGF-1 was significantly increased at both 30PS (p = 0.004) and 30PX (p = 0.038) compared with BL for the Bang® trial. No effect of time for IGF-1 was observed for placebo. Clinical chemistry markers remained within normal clinical ranges for all variables. No differences in hemodynamics were observed between the placebo and Bang® trials.

**Conclusions**

Acute ingestion of Bang® Pre-Workout Master Blaster^TM^ increased lower-body power and endurance as measured by the vertical jump and leg extension repetition tests, respectively. Additionally, Bang® supplementation resulted in potentially favorable serum IGF-1 and HGH responses without adversely affecting clinical safety markers.

**Acknowledgements**

Vital Pharmaceuticals Inc. (VPX) provided the funding for this study.

## P8 Chronic administration of Blamus™, a standardized *Curculigo orchioides* root (black musli) extract, promotes free testosterone level in male rats

### Kanwaljit Chopra^1^, Ravinder Naik Dharavath^1^, Anand Swaroop^2^, Pawan Kumar^3^, Manashi Bagchi^2^, Debasis Bagchi^2,4^

#### ^1^Institute of Pharmaceutical Sciences, Panjab University, Chandigarh 160014, India; ^2^Cepham Research Center, Piscataway, NJ 08854, USA; ^3^Chemical Resources, Panchkula, Haryana 134114, India; ^4^Department of Pharmacological and Pharmaceutical Sciences, University of Houston College of Pharmacy, Houston, TX 77002, USA

##### **Correspondence:** Debasis Bagchi (debasis@cepham.com)

**Background**

*Curculigo orchioides* is an endangered medicinal plant used for diverse medicinal purposes including impotency, aphrodisiac, diuretic, tonic, jaundice, and skin ailments for centuries. Phytochemical investigations of rhizomes revealed the presence of a novel phenolic glycoside, curculigoside, triterpenoid, saponins, flavones, cellulose, hemicellulose, and calcium oxalate. We developed a novel extract of *Curculigo orchioides* (Blamus^TM^, standardized to 30% curculigosides) and assessed its dose- and time-dependent efficacy (0, 10, 25 and 50 mg/kg body weight p.o.) in male rats.

**Methods**

We assessed the body weight, serum free and total testosterone levels in male rats (200-230 g; n = 6) over a period of 28 consecutive days. Moreover, extensive histopathological analyses were conducted on the seminiferous tubules, spermatogenesis, sperm cell morphology, Leydig cells and Sertoli cells.

**Results**

Blamus^TM^ didn’t cause any marked elevation in serum free testosterone levels at either10 or 25 mg/kg body weight doses, however, a 50 mg/kg body weight dose of showed a significant increase in serum free testosterone level (*p < 0.0001). However, no significant increases were observed in serum total testosterone levels at 0, 10, 25 or 50 mg/kg body weight doses of Blamus^TM^. Extensive testicular histopathological analyses including investigations on the seminiferous tubules, spermatogenesis, sperm cell morphology, Leydig cells and Sertoli cells following treatment with either 0, 10, 25or 50 mg/kg body weight doses of Blamus^TM^ were conducted. Results demonstrated dose-dependent improvement in structural integrity. No significant changes were observed in serum SGOT, SGPT, BUN and creatinine levels following treatment with any of the above doses, which demonstrated the broad spectrum safety of Blamus^TM^.

**Conclusion**

The present study demonstrates that Blamus^TM^ may serve as a safe and novel, natural free testosterone booster and provide broad spectrum applications in sports nutrition, muscle building and exercise pathophysiology.

## P9 Protein requirements for optimal nitrogen retention in resistance-trained individuals

### Greg E Popovich (Bradley_g@wvwc.edu)

#### School of Exercise Science & Athletic Training, West Virginia Wesleyan College, Buckhannon, WV 26201, USA

**Background**

The protein requirement of strength-trained individuals has been debated for decades. These debates usually revolve around the disparity between the relatively slow rate of muscle tissue hypertrophy versus the disproportionate amount of dietary protein recommended to achieve the accretion of muscle tissue. One of the most accepted methods of assessing protein status is nitrogen balance. The objective of this study was to statistically analyze previously published nitrogen balance studies to find the model that best describes the relationship between protein intake and nitrogen retention, as well as to elucidate significant variable(s) affecting nitrogen retention.

**Methods**

Nine studies provided a total of 17 subgroups that were allotted various quantities of protein during resistance-training programs. Data were analyzed using Statistica Computer Software. Nitrogen retention was tested for correlations against 10 independent variables using multiple models. Independent variables included protein intake, energy intake, energy balance, average reported daily strength-training duration, lean body mass, and others. The level of significance was set at a value of p ≤ .05.

**Results**

A linear regression model revealed a positive correlation between daily nitrogen intake and nitrogen retention (r = .510) which approached significance (p ≤ .06). The correlation became more pronounced (r = .698) and highly significant (p ≤ .006) once normalized for body mass between nitrogen intake/kg body weight/day and nitrogen balance/kg body weight/day. Zero balance was calculated to occur at a protein intake of 1.35 g/kg/day, and net protein utilization (NPU), equal to the slope, was 27.0%. No other correlations, including total daily training time, reached significance.

**Conclusion**

The greatest predictor of positive nitrogen balance in strength-trained subjects was the amount of protein ingested/kg body weight/day. This reinforces the need to individualize protein intake recommendations based upon body weight. NPU was profoundly decreased on high-protein diets, suggesting a decrease in efficiency of amino acid utilization. The data suggest that resistance-trained persons consume greater than 1.35 g protein/kg body weight/day to optimize muscle anabolism.

## P10 The effect of two different caffeinated energy drinks on resting cardiovascular responses in obese and non-obese trained adults

### Gabriel J. Sanders^1^, Willard Peveler^1^, Corey A. Peacock^2^, Cory Scheadler^1^

#### ^1^Northern Kentucky University, Highland Heights, KY, USA; ^2^Nova Southeastern University, Fort Lauderdale, FL, USA

##### **Correspondence:** Gabriel J. Sanders (sandersg1@nku.edu)

**Background**

Adverse cardiovascular alterations prior to exercise may not be a wholesome indicator of a supplement aimed to enhance performance. Research is inconclusive regarding the impact of energy drinks on cardiovascular measures at rest and comparisons of weight status between energy drinks is absent in the current literature. Therefore, the purpose was to compare two market available energy drinks to a placebo drink and measure heart rate (HR) and blood pressure (BP) at rest in obese and non-obese trained adults.

**Methods**

Twenty-eight (22.1 ± 1.3 years) participants completed the study. Groups were based on BMI, non-obese (n = 20, <30 BMI, 175.7 ± 8.9 cm, 74.1 ± 10.9 kg) and obese (n = 8, >30 BMI, 177.2 ± 14.6 cm, 111.4 ± 21.2 kg). Participants completed three randomized, resting conditions where participants blindly ingested an energy drink (16 oz. Monster Energy ®, 2 oz. 5-hour ENERGY®) or placebo (12 oz. Squirt) then resting in a seated position for one hour. HR and systolic and diastolic BP were measured three times, prior to ingestion of a drink, 30 minutes after and 60 minutes after ingestion. Heart rate and blood pressure were measured with a valid automated blood pressure cuff (OMRON Healthcare INC, Lake Forest, Illinois).

**Results**

A repeated measures ANOVA revealed there was no significant effect of BMI on HR, systolic and diastolic BP (*P* ≥ 0.111). However, there was a significant (*P* ≤ 0.05) time * condition interaction for HR and systolic BP. Post hoc analysis revealed there was a significant (*P* ≤ 0.05) increase from pre to 60 minutes post ingestion for HR and systolic BP for both energy drinks but not placebo (Placebo HR Pre 70.1 ± 2.0 bpm - Post 68.8 ± 1.9 bpm; Monster HR Pre 65.3 ± 2.2 bpm - Post 72.4 ± 2.2 bpm; 5-hour HR Pre 65.8 ± 1.8 bpm - Post 69.6 ± 1.9 bpm) (Placebo Systolic Pre 117.3 ± 1.4 mmHg - Post 116.5 ± 1.9 mmHg; Monster Systolic Pre 114.5 ± 1.6 mmHg - Post 121.3 ± 1.6 mmHg; 5-hour Systolic Pre 113.8 ± 1.4 mmHg - Post 119.8 ± 2.0 mmHg). Moreover, there was a significant difference (*P* ≤ 0.05) in systolic BP between placebo and both energy drinks at 60 minutes with no difference (*P* = 0.393) between the energy drinks at 60 minutes. There were no main or interaction effects of diastolic BP (*P* ≥ 0.290).

**Conclusions**

Energy drinks, and not placebo, increased resting HR and systolic BP after 60 minutes of rest. While there was no difference between the two energy drinks at any time point, both increased systolic BP more than placebo after 60 minutes. Further, BMI status does not appear to influence resting cardiovascular responses to energy drink ingestion. Additional research should assess if energy drinks hamper time to exhaustion biking or running exercise, regardless of BMI.

**Acknowledgments**

This study was funded by the Kentucky Biomedical Research Infrastructure Network (KBRIN) Grant Number 4001095.

## P11 The effects of betaine supplementation on muscle growth and body composition in college aged females

### Andrea Hudson, Taylor Cicholski, Karley Barreno, Kayla Broom, Amanda Cervenka, McKenzie Barch, Mary Frances-Aini, Brandi Washell, Amber Rahman, Raymond Moye, Jason Cholewa

#### Department of Kinesiology, Coastal Carolina University, Conway, SC 29528, USA

##### **Correspondence:** Jason Cholewa (jcholewa@coastal.edu)

**Background**

There is limited research examining chronic (>6 weeks) betaine supplementation in conjunction with resistance training, and currently no research examining the effects of chronic betaine supplementation on changes in muscle growth and body composition in females. The purpose of this study was to investigate the effects of 9 weeks of betaine supplementation on muscle growth and body composition in college aged females.

**Methods**

Young women without prior resistance training experience were recruited for this study. Subjects (N=23; 21.0±1.4 years, 165.9±6.4 cm, 68.6±11.8 kg, 32.7±7.6% body fat) were pair-matched based on body composition and squat strength and then randomly assigned to a placebo (n=12) or a betaine (2.5 g/day, BetaPower®, Finnfeeds Oy, Finland; n=11) group in a double-blind fashion. Training was divided into two lower and one upper body training sessions per week and was performed on non-consecutive days for two 4 week blocks with 1 week of active rest between blocks. Body composition testing (BodPod) was completed pre- and post-study. Thickness of the right rectus femoris was measured using B-mode ultrasound (Chison). Statistical analyses were performed using separate two-way repeated measures ANOVA for each criterion variable with an alpha level of p ≤ .05.

**Results**

There was a significant main effect for time (pre vs. post) for body mass (68.6+11.8 vs. 69.5+12 kg). A significant group x time interaction was found for BF%: BF% decreased significantly more in the betaine group (-3.3+1.9%) compared to placebo (1.7+1.6%). A significant group x time interaction was found for FM: FM decreased significantly more in the betaine group (-2.0+1.1 kg) compared to the placebo group (-.78+1.3 kg). A significant main effect of time was found for FFM (45.4+6.1 vs. 47.9+5.9 kg). A main effect of time was found for MTH (2.97+.54 vs. 3.04+.53 cm). Effect sizes (Cohen’s d) favored betaine compared to placebo for BF% (-.45 vs. -.22), FM (-.22 vs. -.08), and FFM (.48 vs. .31), but not MTH (.26 vs. .24).

**Conclusion**

The results of this study indicate that 9 weeks of betaine supplementation improves body composition by reducing body fat percentage, fat mass, and tended to increase lean body mass in untrained collegiate females. Potential mechanisms for fat reductions and lean mass increases that have been reported in animal models, but require translation to humans, include the suppression of lipogenic (FAS, LPL, FABP) mRNA expression and enzymatic activity, and enhanced IGF-1 signaling pathway, respectively.

**Acknowledgements**

DuPont Nutrition and Health provided the betaine for this study.

## P12 The effects of chronic betaine supplementation on performance in college aged females

### Taylor Cicholski, Andrea Hudson, Karley Barreno, Kayla Broom, Amanda Cervenka, McKenzie Barch, Mary Frances-Aini, Brandi Washell, Amber Rahman, Raymond Moye, Jason Cholewa

#### Department of Kinesiology, Coastal Carolina University, Conway, SC 29528, USA

##### **Correspondence:** Jason Cholewa (jcholewa@coastal.edu)

**Background**

Betaine has been previously shown to enhance force output in men in short-term (2 week) studies. Cholewa et al. (2013) reported increases in lean mass and a trend for greater vertical jump improvements following 6 weeks of betaine supplementation in resistance trained men. There is limited research examining chronic (>6 weeks) betaine supplementation on performance, and currently no research examining the effects of betaine on strength and power performance in females. This study investigated the effects of 9 weeks of betaine supplementation on performance in untrained college aged females.

**Methods**

Young women without prior resistance training experience were recruited for this study. Subjects (N=23; 21.0±1.4 years, 165.9±6.4 cm, 68.6±11.8 kg, 32.7±7.6% body fat) were pair-matched based on body composition and squat strength and then randomly assigned to a placebo (n=12) or a betaine (2.5 g/day, BetaPower®, Finnfeeds Oy, Finland; n=11) group in a double-blind fashion. Training was divided into two lower and one upper body training sessions per week and was performed on non-consecutive days for two 4 week blocks with 1 week of active rest between blocks. Performance testing was conducted pre- and post-training to measure vertical jump, back squat 1RM, and bench press 1RM. Subjects were instructed not to engage in additional exercise nor change their dietary habits during the study. Statistical analyses were performed utilizing separate two-way repeated measures ANOVA for each criterion variable with an alpha level p ≤ 0.05.

**Results**

There were no significant differences between groups for any variables at baseline. There were no significant group x time interactions for any variable assessed. Significant main effects of time (pre vs. post) were found for vertical jump (39.7±6.2 vs. 44.4±7.0 cm), 1 RM back squat (60.1±16.4 vs. 78.2±17.0 kg), and 1 RM bench press (34.9±7.2 vs. 39.0±8.0 kg). Effect sizes (Cohen’s *d*) were similar (betaine vs. placebo) between groups for vertical jump (.79 vs. .75), slightly favored betaine for 1RM back squat (1.19 vs. 1.01), and slightly favored placebo for 1 RM bench press (.46 vs. 69).

**Conclusion**

The results of this study indicate that nine weeks of betaine supplementation does not enhance nor hinder strength and power adaptations during periodized resistance training in previously untrained collegiate females. Differences in supplementation length (2 weeks vs. 9 weeks) or differences in methylation metabolism between genders may explain the discrepancy between the results of this study and previous studies conducted in males.

**Acknowledgements**

DuPont Nutrition and Health provided the betaine for this study.

## P13 Effects of 8 weeks of resistance training and L-citrulline + glutathione supplementation on body composition, muscle mass and performance, and blood clinical safety markers in resistance-trained males

### Paul Hwang^1^, Flor E. Morales Marroquín^1^, Tom Andre^1^, Josh Gann^1^, Caelin Kim^1^, Masahiko Morita^2^, Darryn S. Willoughby^1^

#### ^1^Exercise and Biochemical Nutrition Lab, Baylor University, Waco, TX 76798, USA; ^2^KYOWA HAKKO BIO CO., LTD., Tsukuba, Ibaraki 305-0841, Japan

##### **Correspondence:** Darryn S. Willoughby (darryn_willoughby@baylor.edu)

**Background**

Supplementation of L-citrulline combined with glutathione (GSH) in response to a single bout of resistance exercise has been shown to increase plasma nitric oxide metabolites (NOx) and cyclic guanosine monophosphate (cGMP), which may play a role in muscle protein synthesis. As a result, in response to resistance training (RT) these responses may establish a role for L-citrulline + GSH to serve as an ergogenic aid.

**Purpose**

The primary purpose of the study was to determine the effects of an 8-week RT program in conjunction with L-citrulline + GSH, L-citrulline-malate, or placebo supplementation on body composition and muscle performance. The secondary purpose was to assess the safety of such supplementation protocol by assessing whole blood and serum clinical chemistry markers.

**Methods**

In a randomized, double-blind, placebo-controlled design, 75 resistance-trained males were randomly assigned to ingest 2 g/day of L-citrulline + 200 mg/day of GSH (CIT+GSH), 2 g/day of L-citrulline-malate (CIT), or 2 g/day of cellulose placebo (PLC) daily while also participating in 8 weeks of RT. Participants completed three testing sessions where body composition and muscle performance were assessed before and after 4 and 8 weeks of RT and supplementation. Venous blood samples were obtained before and after 8 weeks.

**Results**

Neither RT nor supplementation had any significant effects on total body mass, total body water, fat mass, muscular strength and endurance, or any of the blood clinical chemistry variables (p > 0.05). However, lean mass increased in both GSH+CIT and CIT compared to PLC, but the increase for GSH+CIT was significantly greater from only PLC after 4 weeks (p < 0.05); however, no further increase existed after 8 weeks (p > 0.05).

**Conclusions**

The supplementation of L-citrulline with GSH during resistance training increases lean mass compared to placebo in resistance-trained males.

**Acknowledgments**

This study was supported by an independent research grant from KYOWA HAKKO BIO CO, LTD., awarded to Baylor University. Dr. Willoughby’s attendance at the conference is being sponsored by KYOWA. Also, Dr. Morita is an employee of KYOWA but he also served as a valuable contributor to the study, and is included as a co-author.

## P14 Effects of chronic l-carnitine supplementation on exercise performance and blood lactate in resistance-trained males

### Majid S. Koozehchian^1^, Amin Daneshfar^2^, Mozhgan Hassanzadeh^3^, Maryam Kaveh^4^, Ebrahim Fallah^2^, Conrad P. Earnest^1,5^, Gholamali Owlia^6^, Mike Greenwood^1^, Richard B. Kreider^1^

#### ^1^Exercise & Sport Nutrition Lab, Human Clinical Research Facility, Department of Health & Kinesiology, Texas A&M University, College Station, TX, 77843 USA; ^2^Department of Kinesiology, Tarbiat Modares University, Tehran, 14115-111, Iran; ^3^Department of Kinesiology, Azad University, Central Branch, Tehran, Iran; ^4^Department of Pharmaceutical Practice, Karnataka College of Pharmacy, Bangalore, India; ^5^Nutrabolt, Bryan, TX, 77807, USA; ^6^Department of Health & Kinesiology, Texas Southern University, Houston, TX, 77004, USA

##### **Correspondence:** Majid S. Koozehchian (majidk@tamu.edu)

**Background**

The purpose of this study was to examine the long-term effects of ingesting L-Carnitine supplement on exercise performance and blood lactate levels.

**Method**

In a randomized, placebo-controlled, double-blind design, 35 male participants (age = 25 ± 2 y; stature = 171 ± 6 cm; body mass = 79.8 ± 8.9 kg; percent body fat = 16.1 ± 5.53%; Control [CON] n = 12, Placebo [PLA] n = 11, L-carnitine [LCR] n = 12) participated in the study. Primary outcomes were total body strength performance, anaerobic performance, and pre- and post-exercise (minutes three, fifteen, and thirty) blood lactate (BL) levels which were measured at baseline and at weeks 3, 6, and 9. All participants were asked to maintain their normal dietary intake during the study period. Participants in both PLA and LCR groups were required to follow a specific resistance training program (4 d/wk, upper body/lower body split) as well as oral ingestion of either PLA or LCR (2 g/day^-1^) for a 9-wk period, while the CON group did not receive any intervention. Data were analyzed by GLM and presented as mean (SD) or change (95% CI).

**Results**

We observed a significant increase in bench press lifting volume (LV) at wk-6 (139 kg, 95% CI 49.1, 230) and wk-9 (238 kg, 95% CI, 132, 343) for LCR. There was a significant improvement in leg press LV at wk-6 (1,483 kg, 95% CI, 543, 2,422) and wk-9 (2,683 kg, 95% CI, 1,568, 3,797) for LCR. The percent change from baseline in LV bench press, leg press, and total strength for LCR was 27.5%, 30.2%, and 15.0% respectively. The significant increase in Wingate mean power (63.4 W, 95% CI 30.5, 96.3) and peak power (239 W/kg, 95% CI 104, 374) was seen at wk-9 for LCR. The percent change from baseline in Wingate mean power and peak power for LCR was 12.8% and 14.4% respectively. The significant BL reduction at wk-9 in 3-min post-exercise (-1.84 mmol/l, 95% CI -2.95, -0.73), 15-min post-exercise (-1.60 mmol/l, 95% CI -2.63, -0.57), and 30-min post-exercise (-0.64 mmol/l, 95% CI, -1.07, -0.21) was observed only in LCR. The percent change from baseline in BL at minutes three, fifteen, and thirty post-exercise for LCR was -17.2%, -14.8%, and -13.6% respectively.

**Conclusion**

Our results indicate that LCR supplementation at the dose of 2 g/day^-1^ increases muscle strength, improves anaerobic performance, and attenuates the blood lactate response to resistance training.

## P15 Impact of chronic l-carnitine supplementation on selected exercise-induced oxidative stress markers in resistance-trained males

### Majid S. Koozehchian^1^, Amin Daneshfar^2^, Mozhgan Hassanzadeh^3^, Maryam Kaveh^4^, Ebrahim Fallah^2^, Conrad P. Earnest^1,5^, Gholamali Owlia^6^, Mike Greenwood^1^, Richard B. Kreider^1^

#### ^1^Exercise & Sport Nutrition Lab, Human Clinical Research Facility, Department of Health & Kinesiology, Texas A&M University, College Station, TX, 77843, USA; ^2^Department of Kinesiology, Tarbiat Modares University, Tehran, 14115-111, Iran; ^3^Department of Kinesiology, Azad University, Central Branch, Tehran, Iran; ^4^Department of Pharmaceutical Practice, Karnataka College of Pharmacy, Bangalore, India; ^5^Nutrabolt, Bryan, TX, 77807, USA; ^6^Department of Health & Kinesiology, Texas Southern University, Houston, TX, 77004 USA

##### **Correspondence:** Majid S. Koozehchian (majidk@tamu.edu)

**Background**

To study the impact of L-carnitine supplementation on selected exercise-induced oxidative stress markers.

**Method**

Thirty-five male subjects (age = 25 ± 2 y; stature = 171 ± 6 cm; body mass = 79.8 ± 8.9 kg; percent body fat = 16.1 ± 5.53%; Control [CON] n = 12, Placebo [PLA] n = 11, L-carnitine [LCR] n = 12) took part in a randomized, placebo-controlled, double-blind study. Oxidative stress markers including total antioxidant capacity (TAC), malondialdehyde (MDA), glutathione peroxidase (GPx), superoxide dismutase (SOD), catalase (CAT), interleukin-6 (IL-6), and tumor necrosis alpha (TNF-α) which were measured at baseline and at weeks 3, 6, and 9. Subjects were required to keep their normal diet throughout the study. Participants in PLA and LCR groups followed a designed resistance training program (4 d/wk, upper body/lower body split) and the ingestion of either PLA or LCR (2 g/day^-1^) for 9 weeks, whereas the CON group received no intervention. All data were then analyzed by GLM and reported as mean (SD) or change (95% CI).

**Results**

There was a statistically significant increase in TAC at wk-9 in LCR (0.18 mmol/L, 95% CI, 0.07, 0.28). A prominent elevation was observed in serum GPx at wk-9 in LCR (1.75 U/ml, 95% CI, 0.61, 2.90). In addition, there was a decrease in serum IL-6 at wk-9 in LCR (-0.53 pg/mL, 95% CI, -1.00, -0.06). The percent change from baseline in serum TAC was increased only in LCR (11.5%), but not in PLA (-0.02%), or CON (-1.94%). There was a decrease in percent change from baseline in serum MDA only in LCR (-31.1%), but not in CON (14.5%). An increase was seen in serum GPx only in LCR (17.4%), nut not in PLA (-3.26).

**Conclusion**

This study indicates that daily consumption of LCR (2 g/day^-1^) improves total antioxidant capacity, while it attenuates exercise-induced oxidative stress makers in resistance-trained males.

## T16 Substantial body recomposition during contest preparation in an experienced female figure competitor: results of 4-compartment model and total body protein calculations

### Grant M. Tinsley (grant.tinsley@ttu.edu)

#### Department of Kinesiology & Sport Management, Texas Tech University, Lubbock, TX, 79424, USA

**Background**

Extremely limited information is available concerning contest preparation in drug-free female physique athletes. Additionally, previous investigations have not employed advanced body composition evaluations, such as the gold-standard 4-compartment model. The purpose of this observational study was to employ advanced body composition assessment methods during the contest preparation phase of an experienced female Figure competitor.

**Methods**

The subject of this case study was a former NCAA Division II athlete who had previously competed in 4 physique competitions. At the commencement of the study, she was beginning an 18-week preparation phase prior to competing in a National Physique Committee (NPC) competition in the Figure division. Throughout the preparation phase, the athlete was closely advised by her coach, a competitive bodybuilder with 20+ years of coaching experience. The athlete meticulously tracked dietary intake, supplement use and exercise sessions. Additionally, the athlete was assessed monthly in the university research laboratories. Assessments included dual-energy x-ray absorptiometry (DXA) and multi-frequency bioelectrical impedance analysis (MF-BIA). Body volume and total body protein were calculated using DXA output as described by Wilson et al. [1], and body composition was assessed using the 4-compartment model as described by Lohman & Going [2]:

Body Volume (L) = 0.933*lean + 1.150*fat + -0.438*BMC + 1.501

Body Protein (kg) = -0.607*volume + 0.623*mass + 0.132*BMC + 0.150*water + 1.076

Fat Mass (kg) = 2.747*volume - 0.714*water + 1.146*BMC - 2.050*body mass

**Results**

Based on 4-compartment calculations, the athlete’s body fat decreased from 18.3% to 12.3% over the first 3 months of preparation. This was due to concomitant decreases in fat mass (12.0 kg to 7.8 kg) and increases in fat-free mass (53.3 kg to 55.2 kg). Additionally, total body protein content increased from 10.4 kg to 10.9 kg. Importantly, substantial differences in body fat percentage between DXA and 4C were observed. Throughout the first 3 months of the preparation period, DXA overestimated body fat percentage by 4.5% on average compared to the 4-compartment model.

**Conclusions**

Substantial body recomposition was demonstrated in a highly-trained female Figure competitor. Concomitant decreases in fat mass and increases in fat-free mass and total body protein took place over the first 3 months of contest preparation. Although DXA is viewed as a superior method of body composition assessment, it substantially overestimated body fat percentage in the observed athlete. When possible, more advanced methods of body composition assessment, such as the 4-compartment model, should be utilized in physique athletes to allow for more accurate evaluation.

**Acknowledgements**

The author has no conflict of interest to report.

**References**

1. Wilson JP, Strauss BJ, Fan B, Duewer FW, Shepherd JA: Improved 4-compartment body-composition model for a clinically accessible measure of total body protein. *The American Journal of Clinical Nutrition* 2013, 97:497-504.

2. Lohman TG, Going SB: Multicomponent models in body composition research: opportunities and pitfalls. *Basic Life Sciences* 1993, 60:53-58.

## P17 The very short-term training model: a reliable model for tracking acute performance adaptions to exercise and nutritional interventions

### M. Travis Byrd, Joel E. Eastman, Haley C. Bergstrom

#### Department of Kinesiology and Health Promotion, University of Kentucky, Lexington, KY, USA

##### **Correspondence:** M. Travis Byrd (mark.travis.byrd@uky.edu)

**Background**

The very short-term resistance training (VST) model, utilizing only 2-3 training sessions, has been shown to increase muscle strength for isometric and isokinetic modalities. This model has been used to examine early phase skeletal muscle, neural, and performance adaptations as well as the efficacy of creatine supplementation to increase strength. Thus, this training model has potential implications for examining acute changes in strength and power from nutritional interventions. No previous studies, however, have applied the VST model to dynamic constant external resistance (DCER) training. Therefore, the purpose of this study was to examine the effect of an upper body DCER exercise (barbell bench press [BP]), using the VST model on strength and barbell velocity.

**Methods**

Ten (5 females, 5 males) subjects (mean ± SD age: 21.4 ± 2.8 yrs; height: 1.75 ± 0.12 m; body mass: 83 ± 8.8 kg) with no resistance training experience within the last three months completed a familiarization visit, two pre-test visits (pre-test 1 and pre-test 2), three training visits, and one post-test visit. For pre-test 1 and pre-test 2, the subject’s 1 repetition maximum (1RM) for the BP was measured as well as the mean (BP_MV_) and peak (BP_PV_) barbell velocities from the BP 1RM. The mean (BT_MV_) and peak (BT_PV_) velocities were also determined from the barbell bench press throw (BT) test, utilizing 35% of the subject’s BP 1RM as resistance. The three training visits consisted of 5 sets of 6 repetitions, at 65% of the subject’s 1RM, with the concentric phase of the BP performed at max barbell velocity. The post-test followed the same procedures as the two pre-test visits. Statistical analyses included one-way repeated measures ANOVAs and paired samples t-tests (alpha level of p≤0.05). The reliability of each variable from pre-test 1 to pre-test 2 was examined using intraclass correlation coefficients (ICC) and standard error of the measurement (SEM).

**Results**

Table 6 shows the mean (±SD) values for pre-test 1, pre-test 2, and post-test for 1RM, BP_MV_, BP_PV_, BT_MV_, BT_PV_, as well as the ICC and SEM values for pre-test 1 to pre-test 2.

**Conclusion**

These findings indicated the strength and velocity variables were reliably measured from pre-test 1 to pre-test 2. In addition, the VST model utilizing an upper body DCER exercise improved strength and barbell velocity in untrained subjects. Thus, the VST model may provide a reliable method for examining acute performance adaptions to exercise and nutritional interventions.

**Table 6 (abstract P17).** Mean ± SD values for pre-test 1, pre-test 2, and post-test as well as the intra-class correlation coefficient (ICC) and standard error of the measurement (SEM) values for Pre-Test 1 and Pre-Test 2 for the subject’s barbell bench press 1 repetition maximum (1RM), the mean barbell velocity from the subject’s 1RM (BP_MV_), the peak barbell velocity from the subject’s 1RM (BP_PV_), the mean velocity of the barbell bench press throw test (BT_MV_), and the peak velocity of the barbell bench press throw test (BT_PV_)

	Pre-test 1	Pre-test 2	Post-test	ICC	SEM
1RM (kg)^a,b^	57 ± 26	58 ± 25	60 ± 26	0.99	2.42
BP_MV_ (m·s^-1^)^a^	0.21 ± 0.10	0.27 ± 0.09	0.34 ± 0.10	0.63	0.06
BP_PV_ (m·s^-1^)^a,b^	0.41 ± 0.11	0.45 ± 0.11	0.54 ± 0.13	0.77	0.05
BT_MV_ (m·s^-1^)^a,b^	0.79 ± 0.11	0.82 ± 0.12	0.84 ± 0.11	0.94	0.03
BT_PV_ (m·s^-1^)^a,b^	1.41 ± 0.22	1.45 ± 0.24	1.50 ± 0.23	0.96	0.05

^a^There was a significant (p < 0.05) increase from Pre-test 1 to Post-test. ^b^There was a significant (p < 0.05) increase from Pre-test 2 to Post-test. There were no significant differences pre-test 1 to pre-test 2 for any of the variables (p > 0.05)

## P18 Estimating body composition at baseline and tracking changes during weight loss: Validity of common methods in comparison to a criterion four-compartment model

### Eric T. Trexler^1,2^, Katie R. Hirsch^1,2^, Malia N. M Blue^1,2^, Meredith G. Mock^1^, Abbie E. Smith-Ryan^1,2^

#### ^1^Applied Physiology Laboratory, Department of Exercise and Sport Science, University of North Carolina at Chapel Hill, NC, USA; ^2^Human Movement Science Curriculum, Department of Allied Health Sciences, University of North Carolina at Chapel Hill, NC, USA

##### **Correspondence:** Abbie E. Smith-Ryan (abbsmith@email.unc.edu)

**Background**

Accurate body composition estimates are valuable for sports nutrition researchers and practitioners performing needs assessments or evaluating the efficacy of interventions. The four-compartment (4C) method utilizes air displacement plethysmography (ADP), bioelectrical impedance spectroscopy (BIS), and dual-energy x-ray absorptiometry (DXA) to estimate body fat percentage (BF%); this 4C method enhances accuracy, but requires greater time, cost, and access to specialized equipment. To facilitate practical body composition assessment and monitoring, the current study compared the validity of ADP, DXA, and amplitude-mode ultrasound (US) for estimating BF% and tracking changes during weight loss in comparison to the 4C criterion method.

**Materials and methods**

Thirty-four overweight/obese male (n=6) and female (n=28) patients (mean ± SD; age: 44.2±12.2 yrs; height: 168.4±8.2 cm; weight: 98.4±26.8 kg; body mass index: 34.6±8.0 kg∙m^-2^) were tested after enrolling in an individualized weight loss program. Participants completed BF% assessments including ADP, BIS, DXA, and US; ADP, BIS, and DXA were used to calculate 4C BF%. Participants were instructed to return for post-testing after losing ≥4.5 kg of body mass; 15 pre/post weight loss pairings were available for analysis. For each method of measurement, validity of baseline BF% estimates and longitudinal changes were evaluated using the Pearson correlation coefficient (r), standard error of estimate (SEE), constant error (CE), and total error (TE) in relation to the criterion 4C measurement. The Bland-Altman method was used to calculate the 95% limits of agreement (95%LA), expressed as ± deviation from CE.

**Results**

For baseline measurements (n=34), BF% was 39.0±8.7% according to the 4C method. ADP estimates showed the highest degree of agreement with 4C BF% estimates (r=0.99, p<0.001; SEE=0.93%fat, CE=1.24%fat, TE=1.54%fat, 95%LA: ±1.81%fat). DXA showed lower agreement with 4C (r=0.92, p<0.001; SEE=3.40%fat, CE=2.57%fat, TE=4.38%fat, 95%LA: ±7.06%fat), and US values showed the least agreement (r=0.68, p<0.001; SEE=6.46%fat, CE=-1.75%fat, TE=6.58%fat, 95%LA: ±12.61%fat). For tracking longitudinal changes (n=15), BF% changed by -3.5±2.8%fat according to 4C estimates. ADP had the highest degree of agreement with 4C (r=0.94, p<0.001; SEE=1.03%fat, CE=-0.24%fat, TE=1.06%fat, 95%LA: ±2.10%fat). DXA values showed less agreement (r=0.58, p=0.03; SEE=2.36%fat, CE=-0.03%fat, TE=2.21%fat, 95%LA: ±4.48%fat), while US estimates displayed lower agreement (r=0.33, p=0.22; SEE=2.74%fat, CE=1.13%fat, TE=2.88%fat, 95%LA: ±5.38%fat).

**Conclusions**

In overweight/obese individuals, ADP provided more valid estimates for cross-sectional and longitudinal changes in body composition in comparison to DXA and US, with DXA performing more favorably than US. Further refinement may be required to facilitate valid, practical body composition assessment using amplitude-mode US in this population.

## P19 Beverage hydration index comparison of enterade®, oral rehydration solution and sports drink

### Kurt J. Sollanek^1^, Matthew Tsurumoto^1^, Sadasivan Vidyasagar^2^, Robert W. Kenefick^3^, Samuel N. Cheuvront^3^

#### ^1^Department of Kinesiology, Sonoma State University, Rohnert Park, CA, USA; ^2^Department of Radiation Oncology, University of Florida Health Cancer Center, Cancer and Genetics Research, Gainesville, FL, USA; ^3^US Army Research Institute of Environmental Medicine, Natick, MA, USA

##### **Correspondence:** Kurt J. Sollanek (sollanek@sonoma.edu)

**Background**

Beverage retention is affected by many factors (e.g., osmolality, electrolytes, etc.). The “Beverage Hydration Index” (BHI) was created to assess the degree to which beverages “hydrate”, by measuring fluid retention after ingesting a 1 liter bolus and comparing it to water. Drinks with carbohydrates and electrolytes score higher on the BHI due to glucose-sodium cotransport at the gut and osmolality approaching isotonicity with blood. Recently, a hypotonic rehydration beverage (enterade®) was developed to take advantage of amino acid-sodium cotransport, thus obviating the need for carbohydrate. The purpose of this investigation was to assess BHI of enterade® in comparison to a carbohydrate-containing sports drink and an oral rehydration solution (ORS).

**Methods**

In a repeated-measures design, forty study participants (males, n=17; females, n=23), age (mean±SD): males 19.7±0.7 y; females 20.3±0.9 y, BMI: males 23.7±2.8; females 22.5±2.7, were studied in a euhydrated state (first morning void USG < 1.025) after an overnight fast. They emptied their bladders, recorded their body mass and then ingested 1 L of fluid over 30 minutes (4 x 250 mL boluses every 7.5 minutes). The beverages, with corresponding osmolalities and kcal content, were as follows: distilled water (~0 mmol/kg; kcals/L), enterade® (195 mmol/kg; 21 kcals/L), ORS (270 mmol/kg; 105 kcals/L) and a sports drink (330 mmol/kg; 237 kcals/m). Each trial was separated by ~1 week. Urine output was collected and measured immediately, and each hour following the first collection for 2 hours, following fluid ingestion. Individual hour cumulative urine mass and BHI were compared by repeated measures one-way ANOVA with a Dunnett’s multiple comparison test to determine which drinks differed from water (P<0.05).

**Results**

Mean (±SD) total urine mass losses over 2 hours for enterade® (1013±288 g) and the ORS (959±234 g) were significantly less than water (1118±242 g; P<0.05) while the sports drink (1075±293 g; P>0.05) was not. The calculated BHI of enterade® (1.15±0.28) and the ORS (1.21±0.28) were greater than water (1.0±0.0; P<0.05) while the sports drink (1.09±0.26; P>0.05) was not.

**Conclusions**

Based upon these data, enterade® and a traditional ORS are superior to water to optimize rehydration, while sports drink was not. Importantly, the high BHI for enterade® was achieved without carbohydrate, making it a low-calorie alternative for effective rehydration.

**Acknowledgements**

This work was supported by Entrinsic Health Solutions, Inc. Authors’ views not official U.S. Army or DoD policy; citations of commercial products are not an endorsement by the DoD.

## P20 Characterization of body composition, blood lipids, and nutrition profile in female healthcare shift-workers when stratified by protein intake

### Alexis A. Pihoker^1^, Meredith G. Mock^1^, Katie R. Hirsch^1,2^, Malia N. M. Blue^1,2^, Kara C. Anderson^1^, Eric T. Trexler^1,2^, Abbie E. Smith-Ryan^1,2^

#### ^1^Department of Exercise and Sport Science, University of North Carolina at Chapel Hill, NC, USA; ^2^Human Movement Science Curriculum, University of North Carolina at Chapel Hill, NC, USA

**Background**

While the ideal macronutrient composition of an individual’s diet is highly debated, data have indicated that high protein (PRO) intake may favorably influence body composition and blood lipid profile. This may be particularly beneficial to shift-workers who have reported higher adiposity. The primary purpose of this study was to evaluate differences in body composition and blood lipids in female hospital shift-workers when stratified by PRO intake. The secondary purpose was to explore the relationships between macronutrient intake, body composition, and blood lipids.

**Methods**

Thirty-three female healthcare shift-workers (Mean ± SD: age = 30.6 ± 9.2 yrs, height = 164.7 ± 6.8 cm, weight = 66.5 ± 10.2 kg, body mass index (BMI) = 24.5 ± 3.7 kg/m^2^) were tested following a minimum eight-hour fast. Dual-energy X-ray absorptiometry was used to measure fat mass (FM), lean mass (LM), bone mineral content (BMC), and body fat percentage (%fat). Blood variables [total cholesterol (TC), high-density lipoproteins (HDL), non-HDL, TC to HDL ratio (TC:HDL), and glucose] were also analyzed. A three-day dietary analysis (2 workdays, 1 off-day) was used to estimate average kilocalorie (kcal), carbohydrate (CHO), PRO, and fat intake. Participants were stratified into two groups using protein intake of ≥ 1.2 g/kg bodyweight (‘adequate’, n=15) or <1.2 g/kg bw (‘deficient’, n=18). Between-group differences were evaluated using a series of independent t-tests. Relationships between dietary intake estimations (kcal, PRO, CHO, and fat) and body composition and blood variables were analyzed using Pearson’s bivariate correlations.

**Results**

The adequate PRO group demonstrated significantly lower bodyweight values [Mean difference (∆)±Std. Error Difference: -10.81±2.89 kg, p=0.001] and significantly lower BMI values (∆=-3.05±1.19 kg/m^2^, p=0.015). The deficient PRO group had significantly higher FM (∆=6.73±2.34 kg, p=0.008), BMC (∆=0.23±0.11 kg, p=0.044), non-HDL (∆=17.09 ± 7.83, p=0.037), and TC:HDL (∆=0.54±0.25, p=0.039). No other body composition or blood variables differed significantly between groups (p=0.053-0.282). There were no significant correlations between dietary variables and blood or body composition variables (R=±0.001-0.326, p=0.064-0.996).

**Conclusions**

Results of the current study demonstrate that meeting an adequate PRO intake is associated with a more favorable bodyweight, fat mass, and blood lipid profile in shift-working females. This indicates that surpassing general PRO recommendations (0.8 g/kg) may advantageously influence body composition and blood lipid profile. However, the lack of significant relationships between dietary variables and body composition and blood measurements suggests that macronutrient intake may be one of many factors contributing to body composition and blood lipids in shift-working personnel.

## P21 The effects of protein supplementation on body composition and metabolic rate changes following bariatric surgery

### Katie R. Hirsch^1,2^, Malia M. N. Blue^1,2^, Eric T. Trexler^1,2^, Alexis A. Pihoker^1^, Shawn Ahuja^1^, Kara C. Anderson^1^, Meredith G. Mock^1^, Abbie E. Smith-Ryan^1,2^

#### ^1^Applied Physiology Laboratory, Department of Exercise and Sport Science, University of North Carolina, Chapel Hill, NC, USA; ^2^Human Movement Science Curriculum, Department of Allied Health Science, University of North Carolina, Chapel Hill, NC, USA

##### **Correspondence:** Abbie E. Smith-Ryan (abbsmith@email.unc.edu)

**Background**

Over two-thirds of the US population is considered overweight/obese and at an increased risk for various metabolic diseases. Bariatric surgery has emerged as an effective and sustainable weight loss option, resulting in significant loss of fat mass (FM). However, substantial decreases in fat free mass (FFM) and resting metabolic rate (RMR) are also common, increasing chances of weight regain. Increased consumption of dietary protein has been shown to attenuate losses in FFM during weight loss, which can help maintain RMR. It is currently recommended that bariatric surgery patients consume at least 60 g of protein per day, yet previous research suggests that many bariatric patients have insufficient protein intake following surgery. Therefore, the purpose of this study was to evaluate the effects of daily protein supplementation on changes in body composition and RMR following bariatric surgery.

**Methods**

A pilot sample of ten bariatric surgery patients (Mean ± SD; age=43.1±11.1 yrs; BMI=55.0±15.1 kg·m^-2^; body fat percentage [%BF]=43.2±6.3%) from an ongoing, randomized, controlled intervention, completed two visits: one prior to surgery and another approximately three weeks post-surgery. Multi-frequency bioelectrical impedance spectroscopy was used to evaluate FM, FFM, %BF, and total body water (TBW). Portable indirect calorimetry was used to measure RMR. Patients were randomly assigned to either protein supplementation (PRO; n=5), consuming provided protein shakes for three weeks, or standard of care (SOC; n=5), following physician-nutritionist team recommendations.

**Results**

Changes between treatment groups were not significantly different (p>0.05), but across the entire group there were significant decreases in weight (Mean change [Δ] ± SD= -10.1±6.5 kg), FFM (Δ= -8.0±6.0 kg), and TBW (Δ= -5.2±3.7 L) (p<0.05) three-weeks post-surgery; there were no significant changes in %BF (Δ= 1.4±3.1%), FM (Δ= -2.1±5.3 kg), or RMR (Δ= -292.7±582.2 kcal·d^-1^) (p>0.05). Although non-significant, RMR decreased less with PRO (Δ= -166.4±576.8 kcal·d^-1^) than with SOC (Δ= -419.0±624.6 kcal·d^-1^).

**Conclusion**

In as little as three weeks following bariatric surgery, there were significant decreases in body weight, primarily associated with losses in FFM, specifically, loss of body water. Although changes in body composition and metabolic rate were not significantly different between treatment groups, the SOC group had a greater decrease in RMR compared to the PRO group. Although this is a small pilot sample, results suggest that protein supplementation following bariatric surgery has the potential to help prevent decreases in RMR that might otherwise lead to future weight regain.

**Acknowledgements**

This project was supported by Premier Nutrition Inc.

## P22 Individualized hydration plans improve performance outcomes during prolonged, hard training sessions in NCAA Division I and II student athletes

### David Ayotte Jr., Michael Corcoran

#### Merrimack College, North Andover, MA, USA

##### **Correspondence:** Michael Corcoran (corcoranm@merrimack.edu)

**Background**

The purpose of this experimental, randomized cross-over study was to determine whether tailored hydration plans improve athletic performance in collegiate athletes during hard training sessions. The rationale for this study is based on previous research, which indicates that athletes commonly consume insufficient fluid and electrolytes before, during, and after training sessions. Athletes who engage in frequent rigorous and prolonged training sessions, may require hydration plans tailored to their individual physiology to improve their performance.

**Methods**

Fifteen varsity student athletes from Merrimack College aged 20 ±0.85 years were recruited. Athletes were eligible to participate if they were injury free and could exercise at ≥75% of their maximum heart rate for at least 45 minutes. Informed consent was obtained from the athletes, coaches and athletic training staff and all methods were approved by the college institutional review board. After completing a questionnaire assessing hydration knowledge, each athlete underwent an assessment of their sweat rate through participating in a high intensity training bout lasting at least 45 minutes. Bodyweight loss was determined and adjusted for fluid consumption during training. Athletes were randomized to a prescription hydration plan (PHP) or instructed to follow their normal hydration habits (NHP) during training. The PHP was developed by assessing sodium loss through sweat using the Precision Hydration Sweat Analysis software. A hydration plan was recommended to each participant based off of the results of their individual sweat composition and sweat rate. Reaction time using Neurotracker technology and lower body power through standing long jumps were assessed before and immediately after an intense training session where participants in both groups exercised at ≥75% of their max heart rates for at least 45 minutes. During training sessions, heart rate was monitored remotely by Zephyr technology to gauge exertion level and recovery. After a washout period of 7 days, the PHP group repeated the training bout with their normal hydration routine, while the NHP group completed the protocol following their PHP plan.

**Results**

Compared with following their NHP, participants following their PHP jumped 2.13 ± 3.15 inches farther (*P*<0.05), tracked objects 0.33 ± 0.33 meters per second quicker (*P*<0.05) and experienced a faster drop in heart rate (3.0 bpm) five minutes post practice.

**Conclusion**

A tailored hydration plan, based on fluid and sodium loss, mitigated the decline in performance observed during intense practices of 45 minutes or longer in duration and has the potential to significantly improve athletic performance.

**Acknowledgements**

We are grateful to the athletic care team and coaches for providing assistance with recruitment for this study and ensuring the safety and welfare of these athletes while engaging in these training sessions. We also are grateful to the athlete participants themselves for their willingness to volunteer.

## P23 A randomized double blind placebo controlled clinical trial evaluating the effects of an investigational study product on exercise induced muscle soreness, markers of inflammation, muscle damage and exercise performance in healthy males

### Douglas Kalman^1,3^, William Howitt^1^, Cassandra Holms^1^, Susan Hewlings^2,3^

#### ^1^QPS-BKCA, Springfield, MO, USA; ^2^Department of Human Environmental Studies, Central Michigan University, Mt. Pleasant MI, USA; ^3^Substantiation Sciences, Weston, FL, USA

##### **Correspondence:** Douglas Kalman (douglas.kalman@qps.com)

**Background**

Gherkin extract, a type of cucumber, is a pure botanical extract that has been shown to have anti-inflammatory and pain relieving properties in observational studies in humans. The purpose of the study was to determine the effects of a proprietary cucumber extract on [active test product 150 mg Gherkin (*Cucumis sativus L.)*] known in the US as Cuvitus™ (known in Europe as Actido^®^): perceived levels of muscle soreness/discomfort after exercise; markers of inflammation; exercise performance, and exercise performance maintenance.

**Methods**

In a randomized double blind placebo design twenty-four healthy male subjects were randomly assigned to take either placebo or 1 capsule in the morning and 1 capsule in the evening (150 mg each) of Cuvitus for 6 days prior to attending their first of two exercise sessions. Prior to each exercise session subjects were given the Visual Analog Pain/Discomfort Scale (VAS), vital signs and blood was drawn and analyzed for TNF-alpha, IL-6, IL-10, IL-1 beta. Subjects then received Cuvitus or placebo 1 hour before a leg extension exercise designed to induce muscular soreness, cellular damage and to assess performance. Tests and blood draw were repeated one hour post exercise. Subjects returned 7 days later to repeat the exercise session.

**Results**

There were significant (p<.05) changes in TNF alpha within groups for both placebo and control, but no significant between group differences. There were significant improvements and enhanced recovery of IL-6 within group for each group with improvements trending toward being significantly greater in the treatment group compared to placebo (p=0.0581). There were significant changes in IL-10 within each group. However the acute recovery was significantly better in the treatment group as compared to placebo for the 1-2 hours post (p< 0.0423). The rise in IL-1 beta trended towards being significantly greater in the placebo compared to the treatment (p=0.058). DOMS was significantly greater post exercise compared to pre-exercise in both groups. The treatment resulted in a 6.2 times improvement in exercise performance over placebo, which trended toward a significantly greater gains in the treatment group compared to placebo.

**Conclusion**

Taking Cuvitus 150 mg twice a day for 2 weeks lead to better performance when compared to Placebo as well as improvements in anti-inflammatory and immune supportive markers.

**Disclosures**

QPS, a Contract Research Organization received a research grant from XSTO Solutions to execute this clinical trial.

## P24 Estradiol, but not fish oil supplementation, may attenuate eccentric exercise-induced muscle damage in females

### Sarah K. McKinley-Barnard^1^, Thomas L. Andre^2^, Josh J. Gann^2^, Paul S. Hwang^2^, Darryn S. Willoughby^2^

#### ^1^Department of Health, Kinesiology, and Sport; University of South Alabama, Mobile, Alabama; ^2^Exercise and Biochemical Nutrition Laboratory, Department of Health, Human Performance, and Recreation; Baylor University, Waco, Texas

##### **Correspondence:** Darryn S. Willoughby (darryn_willoughby@baylor.edu)

**Background**

Due to the supposed cyto-protective effects of estradiol (estrogen), females are thought to be less predisposed to exercise-induced muscle damage (EIMD) than males, but may be more prone to muscle damage during the low estrogen point in their 28-day cycle (follicular phase) compared to their high estrogen point (luteal phase). It has also been theorized that estradiol may have the functional capacity to act as a membrane stabilizer, thereby attenuating release of muscle damage markers post-exercise. Fish oil supplementation has also been suggested to be important for cyto-protection due to its anti-oxidant potential for significantly decreasing markers of muscle damage. The anti-inflammatory and anti-oxidative properties of omega-3 fatty acids has been hypothesized to help counteract the inflammatory state associated with EIMD.

**Purpose**

The purpose of this study was to compare two phases of the menstrual cycle to determine whether the difference in estradiol levels, as well as determine if fish oil supplementation, would attenuate EIMD following a bout of eccentric exercise.

**Methods**

In this double-blind study, 22 physically-active females were randomly assigned to ingest either 6 grams of fish oil or a placebo daily for 21 days. Participants underwent an eccentric exercise bout of the knee extensors on two occasions, during the mid-follicular (MF) phase (day 6) and mid-luteal (ML) phase (day 21) of the 28-day menstrual cycle. Prior to (PRE), at 6 (6HRPOST), and 24 hours post-exercise (24HRPOST) for each session, participants’ muscle strength was assessed, and venous blood samples and muscle biopsies were obtained. Data were analyzed utilizing a 2 x 2 x 3 repeated measures MANOVA for each criterion variable (*p* ≤ .05). Further analysis of the main effects for Test was performed by separate ANOVAs.

**Results**

Delayed onset muscle soreness was significantly greater at the 6HRPOST and 24HRPOST time points compared to the PRE (*p* < .0001). Superoxide dismutase, tumor necrosis factor-α, and nuclear factor-κB p65 concentrations were all significantly higher at the MF phase compared to the ML phase (*p* < .001, *p* = .05, *p* = .04, respectively). There were no statistically significant differences observed for other criterion variables.

**Conclusion**

The results of this study demonstrate that estradiol, but not fish oil supplementation, may exert a cyto-protective effect on the sarcolemma, thereby protecting skeletal muscle from EIMD. The results further suggest that higher levels of estradiol may even provide additional protection against EIMD during the ML phase of the female menstrual cycle.

## P25 Dose-dependent improvement of body composition after supplementation of specific bioactive collagen peptides in combination with resistance exercise

### Steffen Oesser^1^, Denise Zdzieblik^2^, Daniel König^1^

#### ^1^CRI, Collagen Research Institute, Kiel, Germany; ^2^Department of Sport and Sport Science, University of Freiburg, Freiburg, Germany

##### **Correspondence:** Steffen Oesser

**Background**

Several investigations have shown that the combination of resistance exercise and protein supplementation increases fat free mass (FFM) and muscle strength, and could also reduce fat mass (FM). Although the concept of protein supplementation is generally accepted, the optimal type and amount of protein is still under discussion. The efficacy of collagen peptide intake in improving the body composition was recently demonstrated in an RCT on sarcopenic men [1]. Due to their excellent bioavailability and positive impact on connective tissue metabolism [2], bioactive collagen peptides (BCP) might be interesting as a supplement in sports nutrition.

**Materials and methods**

The effect of post-exercise supplementation of specific BCP (BODYBALANCE®) on FFM and FM was tested on 167 men aged from 30 to 60. The study participants underwent 60 minutes of resistance training three times weekly and were treated with 15 g BCP or a placebo for 12 weeks. In addition, a daily BCP dosage of 10 g and 20 g was tested. Changes in FFM and FM were measured by DEXA scans at the beginning of the study and after 12 weeks. Differences within the groups were analyzed with a Wilcoxon Rank-Sum test, and changes between the study groups were tested using the Mann-Whitney U-test.

**Results**

The results revealed a significant (p<0.05) increase in FFM after BCP supplementation of 15 g/day compared to placebo. FFM gain was more than 80% higher than in individuals who only did the training. In addition, FM was significantly (p<0.05) reduced after BCP supplementation by 1.8 kg compared to placebo. A daily dosage of 10 g and 20 g BCP intake also led to a pronounced statistically significant (p<0.05) FFM increase and FM reduction respectively compared to the baseline data. Although no significant differences between the study groups could be determined, the effect size (Cohen’s d) clearly indicated a dose-dependent effect. For FFM changes, the effect size increased from d=0.380 for 10 g BCP to d=0.433 for 15 g BCP, and d=0.510 for 20 g BCP intake. The effect size for FM loss increased concurrently from d=0.402 (10 g BCB) to d=0.459 (20 g BCP).

**Conclusions**

The results show that BCP supplementation combined with resistance training had a positive effect on body composition, as indicated by an increased FFM and a more pronounced FM reduction. On the basis on these results, BCP appear to offer an interesting supplement for optimized sports nutrition. The current results suggest an optimal dosage of 15 g per day.

**Acknowledgements**

The studies were conducted with the approval of the Ethics Committee of the Medical Faculty of the University of Freiburg. All participants gave written informed consent.

**Trial registration**

The RCT is registered in the German Clinical Trial Register with its ID-No. DRKS00008925.

**References**

1. Cermak NM, Res PT, de Groot LCPGM, *et al.* (2012) *Am J Clin Nutr* **96**,1454–64.

2. Zdzieblik D, Oesser S, Baumstark MW *et al. K* (2015) *Br J Nutr* **114** 1237–45.

## P26 The influence of training background on endogenous antioxidant responses to acute aerobic or anaerobic exercise

### Brittany N. Bozzini, Joseph K. Pellegrino, Alan J. Walker, Chris E. Ordway, Anthony Poyssick, Sean P. Conway, Peter J. Gillies, Shawn M. Arent

#### IFNH Center for Health and Human Performance, Rutgers University, New Brunswick, NJ, 08901, USA

**Background**

Various antioxidant systems are activated to aid in management of reactive oxygen species (ROS) production during exercise. The purpose of this study was to examine the effect of training background on markers of oxidative stress and endogenous antioxidant activity resulting from either aerobic or anaerobic exercise bouts.

**Methods**

Participants (N=40) were split into two equal groups based on training background (endurance (END) or resistance (RES) trained) with 10 males and 10 females in each (END=23.3±4.1y, 65.2±4.1 kg; RES=22.7±2.5y, 69.2±7.2 kg). On separate days, individuals performed either 45-minute aerobic (AE) or weight-training (WT) exercise bouts. Serum was collected prior to, immediately after, and 60 min post exercise (T_0_, T_1_ & T_2_), then analyzed via UHPLC/MS using a metabolomics panel by Metabolon for identification of biochemicals implicated in ROS-scavenging pathways. RM-ANOVAs were conducted with significance set at P<0.05. Heat maps and magnitude of change from T_0_ were generated for the metabolomic response.

**Results**

From T_0_ to T_1_, cysteine-glutathione disulfide concentration significantly increased during WT only (END-WT_T0-T1_=1.2±0.2-fold; RES-WT_T0-T1_=1.4±0.6-fold; P<.05). However, by T_2_ both AE and WT elicited a significant increase from T_0_ (AVG_T0-T2_=1.7±0.5-fold; P<.05). Training background and exercise session appear to influence cystathione concentrations. During AE, both RES and END experienced significant increases for cystathione from T_0_ to T_1_ (END-AE_T0-T1_=1.4±0.6-fold; RES-AE_T0-T1_=1.5±0.8-fold; P<.05), but differed from T_1_ to T_2_. A significant rise continued in RES-AE (RES-AE_T1-T2_=1.3±0.6-fold; P<.05), while no significant changes were depicted in END-AE at T_2_. Following the WT session, a significantly increased cystathione concentration was observed in RES from T_0_ - T_1_ (RES-WT_T0-T1_=1.4±0.6-fold; P<.05) and remained elevated through T_2_ (RES-WT_T0-T2_=1.7±1.0-fold; P<.05), while END demonstrated no significant changes. Across all groups and conditions, heme and plasmalogen ROS-scavenging pathways displayed increased activity during exercise, but returned to baseline by T_2_. Glutathione and taurine metabolism remained active throughout recovery. No sex differences were identified.

**Conclusion**

Our results revealed that continued endogenous antioxidant activity post-exercise attenuated ROS damage to lipid membranes and RBC’s seen during exercise. Findings indicate WT elicited a more robust up-regulation of ROS-scavenging than AE, specifically within glutathione metabolism. This may have caused an augmented ability to handle exercise-induced oxidative stress in RES compared to END individuals regardless of acute exercise modality. Sustained activity of glutathione and taurine production post-exercise highlights the importance of cysteine and cystathione metabolism in antioxidant capacity, as they are precursors to both glutathione and taurine.

**Acknowledgements**

Funding provided by NJ Institute for Food, Nutrition, & Health.

## P27 Aerobic or resistance exercise bout effects on markers of the endocannabinoid system and tryptophan metabolism: The role of training emphasis

### David J. Sanders, Joseph K. Pellegrino, Christopher E. Ordway, Alan J. Walker, Anthony N. Poyssick, Sean P. Conway, Peter J. Gillies, Shawn M. Arent

#### IFNH Center for Health and Human Performance, Rutgers University, New Brunswick, NJ, 08901, USA

**Background**

Exercise yields numerous acute physiological responses that affect many variables, including mood. The endocannabinoid system (ECS) and its ligands are associated with pain-relief, sedation, anxiolysis, and well-being. Tryptophan (Trp) and its derivative serotonin (5-HT) have also been shown to contribute to feelings of well-being and happiness. The purpose of this study was to determine the effect of an aerobic cycling or anaerobic weight-training exercise bout on markers of the ECS and Trp metabolism.

**Methods**

Participants (N=40) were equally distributed into one of 2 groups based on training background (endurance (END) or resistance (RES) trained) (END: M_age_ = 23.3±4.1y, M_height_ = 1.7±0.1 m, M_weight_ = 65.2±6.6.1 kg, M_%BF_ = 16.7±8.2%; RES: M_age_ = 22.7±2.5y, M_height_ = 1.7±0.1 m, M_weight_ = 69.3±11.3 kg, M_%BF_ = 19.4±6.6%). Participants performed a 45-minute aerobic (AE) or weight-training (WT) exercise condition on separate days. Serum was collected before, immediately after, and 60-min post-exercise (T_0_, T_1_, and T_2_, respectively). Samples were analyzed via UHPLC/MS by Metabolon using a metabolomics platform for identification of oleoyl ethanolamide (OEA), palmitoyl ethanolamide (PEA), linoleoyl ethanolamide (LEA), Trp, and 5-HT. RM ANOVAs were conducted with significance set at P<0.05. Heat maps and magnitude of change were generated for the metabolomic response.

**Results**

From T_0_ to T_1_, OEA (END=1.6±0.3-fold, RES=1.6±0.4-fold), PEA (END=1.4±0.3-fold, RES=1.4±0.3-fold), and LEA (END=1.7±0.5-fold, RES=1.7±0.5-fold) increased significantly (P<0.05) and similarly during the AE session in both groups. From T_0_ to T_1_, OEA (END=1.2±0.3-fold, RES=1.2±0.2-fold), PEA (END=1.2±0.3-fold, RES=1.2±0.2-fold), and LEA (END=1.1±0.4-fold, RES=1.1±0.4-fold) increased during WT in both groups (P<0.05). The ECS ligands remained elevated from baseline at T_2_ in both groups and exercise trials. From T_0_ to T_1_, Trp went unchanged (END=1.0±0.2-fold) or marginally decreased (RES=0.9±0.1-fold), and 5-HT significantly increased (END=1.1±0.4-fold, RES=1.1±0.2-fold) during the AE session (P<0.05). Similar findings were observed during WT in Trp (END=1.0±0.2-fold, RES=0.9±0.2-fold) and 5-HT (END=1.0±0.3-fold, RES=1.2±0.2-fold). Trp remained unchanged at T_2_ in both groups and both exercise trials. 5-HT decreased from AE (END=0.7±0.4-fold, RES=0.8±0.3-fold), and from WT (END=0.8±0.4-fold, RES=0.7±0.3-fold) by T_2_.

**Conclusions**

Aerobic exercise, regardless of training background, appears to elicit a modestly greater response from the ECS than weight-training. This may occur because of the greater contribution of lipid metabolism to aerobic activity than anaerobic activity. Thus, the ECS response may be partially dependent upon substrate metabolism. Also, increases in 5-HT were less than that of ECS ligands. Further examination of the relative responses in these systems may help elucidate mechanisms driving certain psychological responses with different exercise modalities.

**Acknowledgements**

Funding provided by NJ Institute for Food, Nutrition, & Health.

## P28 Glucose utilization and ketone body production after differing exercise modalities in aerobically or anaerobically trained males and females

### Bridget A. McFadden, Joseph K. Pellegrino, Alan J. Walker, Christopher E. Ordway, Sean P. Conway, Anthony N. Poyssick, Peter J. Gillies, Shawn M. Arent

#### IFNH Center for Health and Human Performance, Rutgers University, New Brunswick, NJ, 08901, USA

##### **Correspondence:** Bridget A. McFadden

**Background**

Resistance and endurance exercise elicit unique acute physiological responses, which lead to chronic adaptations depending on an individual’s training background. Fuel utilization as it relates to the metabolism of ketone bodies as an alternative or complementary fuel source to glucose is one such response. The purpose of this study was to examine the effects of resistance or endurance exercise on glucose metabolism and ketone body production in differentially trained males and females.

**Methods**

Subjects (n=40) were equally distributed into one of 4 groups (n=10) based on sex (Age_M_=24±4y, Weight_M_= 72.8±7.8 kg, %BF_M_=12.8±5.7%; Age_F_=22±2y, Weight_F_=61.5±7.2 kg, %BF_F_= 23.3±4.8%) and training history (endurance-trained [END] or resistance-trained [RES]). All subjects engaged in 45-min aerobic (AE) or weight-training (WT) exercise bouts which were performed on separate days. Time of day and pre-exercise nutrition were controlled for each participant. Serum was collected pre, 0 and 60-min post-exercise (T0, T1 & T2), and analyzed via UHPLC/MS using a metabolomics panel by Metabolon for identification of glucose [glu], pyruvate [pyr], lactate [lac], acetoacetate [AcAc] and 3-hydroxybutyrate [BHBA]. RM-ANOVA’s were conducted on log-transformed-median-scaled outputs with significance set at P<0.05. Heat-maps and fold-of changes were generated from the metabolomics response.

**Results**

[Glu] increased (1.23±0.09-fold; P<0.05) at T1 before returning to baseline at T2 in all conditions except END-WT-females and RES-AE-males, in which no changes from baseline were observed. [Pyr] and [lac] followed the same pattern across all conditions: roughly a 3-fold (3.70±0.86; P<0.05) increase at T1 followed by a modest decline (0.43±0.04-fold; P<0.05) by T2. Ketones presented a clear differential response to training background and sex. During all WT-conditions, ketones remained unchanged from baseline regardless of sex or training status. Changes were only observed during AE-conditions. Across females performing AE, ketones increased (2.74±0.46-fold; P<0.05) from T0–T1 and remained elevated. In RES-males performing AE, ketones increased (3.72±0.04-fold; P<0.05) and remained elevated at T2; however, END-males saw no change in ketones from T0-T2 during AE.

**Conclusion**

It appears WT failed to elicit changes in ketone mobilization from baseline. Ketones had a consistent rise following AE and remained elevated 60-min post, except for male-END-AE, where both AcAc and BHBA returned to baseline. It seems AE energetically favored fuel flexibility, and END-males were better equipped to handle this stressor than their RES or female counterparts. The lack of any change in [glu] for END-females during WT and RES-males during AE may be a result of the novelty of the exercise.

**Acknowledgements**

Funding provided by the NJ Institute for Food, Nutrition, & Health.

## P29 Two years on a high-protein diet: much ado about nothing

### Anya Ellerbroek, Corey Peacock, Tobin Silver, Jose Antonio

#### Department of Health and Human Performance, Nova Southeastern University, Davie FL, USA

##### **Correspondence:** Jose Antonio (ja839@nova.edu)

**Background**

The consumption of a high protein diet (>3 g/kg/d) over a one-year period in highly trained men has been shown to have no harmful effects on kidney and liver function. Thus, the purpose of these case reports was to do a follow-up investigation of five subjects on a high protein diet over another 1-year period.

**Methods**

Five healthy resistance-trained men (mean ± SD; age 30 ± 5 yr; height 177.9 ± 5.5 cm) volunteered to continue to consume a high-protein diet (>2.2 g/kg/d over another 12 month period). Subjects came to the lab every 6 months to assess body composition. Subjects continued to provide dietary self-reports via the Myfitnesspal app (>150 diet recalls per year). No other instructions were given. Each subject was provided with protein powder so they could attain their protein intake goals. A comprehensive metabolic panel and blood lipid panel was assessed in a fasted state.

**Results**

Please see Tables 7, 8 and 9.

**Conclusion**

Consuming a high-protein diet for 2 years in resistance-trained men has no deleterious effects on liver or kidney function. Subjects also demonstrated above average bone mineral density.

**Acknowledgements**

Protein was provided by Dymatize. This study was unfunded.

**Table 7 (abstract P29).** Protein Intake

	Age	Baseline PRO (g)	g/Kg/d	Year 1: PRO (g)	g/Kg/d	Year 2: PRO (g)	g/Kg/d
Subject 1:	25 yr	138	1.5	217	2.2	255	2.6
Subject 2:	26 yr	193	2.7	278	3.4	285	3.6
Subject 3:	30 yr	395	4.0	524	5.1	562	5.8
Subject 4:	31 yr	184	2.2	250	3	222	2.7
Subject 5:	38 yr	163	2.0	198	2.5	200	2.6

Data are expressed as a yearly mean protein intake. Legend: yr- years, PRO-protein, g-grams, kg-kilogram, d-day

**Table 8 (abstract P29).** Select Clinical Measures

	Baseline	Year 1	Year 2	Reference
Glucose mg/dL	83±6	79±2	86±4	65-99 mg/dL
BUN mg/dL	24±6	21±8	24±8	7-25 mg/dL
Creatinine mg/dL	1.17±0.42	1.11±0.47	1.24±0.18	0.60-1.35 mg/dL
eGFR	97±27	102±26	95±28	>OR=60 mg/dL
AST U/L	31±8	27±5	28±4	10-40 U/L
ALT U/L	29±12	28±11	26±7	9-46 U/L

Data are mean ± SD. There were no significant differences between groups. ALT: alanine transaminase, AST: aspartate transaminase, BUN: blood urea nitrogen, eGFR: estimated glomerular filtration rate, g: grams, L: liter, and mg: milligrams

**Table 9 (abstract P29).** Bone Mineral Density

	Subject 1	Subject 2	Subject 3	Subject 4	Subject 5
T-score	2.6	0	2.6	0.8	1.2
Normal T-score	-1	-1	-1	-1	-1
Low bone mass T-score	btw.-1.0 and-2.5	btw.-1.0 and-2.5	btw.-1.0 and-2.5	btw.-1.0 and-2.5	btw.-1.0 and-2.5
Osteoporosis T-score	≤-2.5	≤-2.5	≤-2.5	≤-2.5	≤-2.5

## P30 Changes in nutritional biomarkers, perceived stress, and performance in D1 female soccer players across a competitive season

### Morgan L. Hofacker, Alan J. Walker, Bridget A. McFadden, David J. Sanders, Anthony N. Poyssick, Nicholas S. Mackowski, Christopher E. Ordway, Marissa L. Bello, Brittany N. Bozzini, Shawn M. Arent

#### IFNH Center for Health and Human Performance, Rutgers University, New Brunswick, NJ, 08901, USA

**Background**

A balance between training stress and recovery is essential for successful athletic performance, thus the development of an evidence-based approach to monitoring changes in stress and recovery is critical. The purpose of this study was to combine analysis of nutritional biomarkers with mood, sleep, and performance assessments to examine changes in recovery and training status throughout a competitive season.

**Methods**

Division I female collegiate soccer players (N=25; M_age_=19.4 ± 1.4 yrs; M_weight_ = 66.1 ± 1.3 kg) participated in blood draws at the beginning of preseason (T1) and every four weeks after within ~18 hours following a game (T2-T4). Athletes arrived euhydrated following an overnight fast. Glutamine (Gln), Taurine (Tau), Tryptophan (Trp), Phenylalanine (Phe), Iron (Fe), Vitamin B12 (VitB12), Vitamin D (VitD), and Omega-3 (OMG3) were analyzed. Mood, sleep and vertical jump (VJ) were also assessed. Mood and sleep were assessed using the Multi-Component Training Distress Scale (MTDS), and the Pittsburgh Sleep Quality Index (PSQI), respectively. RM MANOVAs with univariate follow-ups were conducted with significance at P<0.05.

**Results**

Gln increased from T1-T2 (∆Gln = 82.1 ± 23.1 umol/L, P<.05) then returned to baseline. Trp decreased from T2-T3 (∆Trp = -10.9 ± 4.3 umol/L, P<.05) and remained depressed. There were no significant changes in Phe or Tau. Fe decreased from T1-T2 (∆Fe= -29.6 ± 7.9 mcg/dL, P<.05), before returning to baseline. VitB12 increased from T1-T3 (∆VitB12= 72.0 ± 18.8 pg/mL, P<.05) and remained elevated. VitD decreased from T1-T2 (∆VitD= 6.8 ± 1.4 ng/mL, P<.05) and continued a downward trend. OMG3 increased from T1-T2 (∆OMG= 0.5 ± 0.1%, P<.05), then returned to baseline. Total mood disturbance increased from T2-T3 (∆Mood= 6.4 ± 1.9, P<.05) and remained elevated. No changes in sleep quality (SQ) were seen. Sleep duration (SD) increased from T3-T4 (∆SD=0.4±0.1, P<.05). VJ was maintained from T1-T3 but began to decline at T4.

**Conclusions**

Biomarker changes appear to coincide with mood and performance changes throughout a season. Trp levels declined through T4 as mood disturbance increased. Trp, a precursor of serotonin, may provide a mechanism for understanding changes in mood typically reported with overreaching. VitD decreased throughout the season, and these changes preceded performance changes. SQ may be more important for full recovery than SD, as increased SD did not mitigate mood or VJ changes. Changes in nutritional biomarkers appear to occur before or in conjunction with psychological or performance changes and could serve as early indicators for overreaching.

**Acknowledgements**

Funding provided by Sports and Human Performance Diagnostics of Quest Diagnostics.

## P31 Self-reported anger measurements of non-medical anabolic-androgenic steroid users

### Guillermo Escalante^1^, Rick Collins^2^, Jack Darkes^3^, Jason Cohen^4^

#### ^1^Department of Kinesiology, California State University, San Bernardino, CA, USA; ^2^Collins Gann McCloskey & Barry PLLC, Mineola, NY, USA; ^3^Department of Psychology, University of South Florida, Tampa, FL, USA; ^4^Licensed Clinical Psychologist, San Luis Obispo, CA, USA

##### **Correspondence:** Guillermo Escalante (gescalan@csusb.edu)

**Background**

Controversy exists regarding the relationship of anabolic-androgenic steroid (AAS) use and anger. This investigation examines self-reported anger measurements among AAS users before, during, and after AAS use.

**Methods**

A total of 20.9% (n=408) of 1955 male non-medical anabolic-androgenic steroid (NMAAS) users (age = 31.1 +/- 9.2 years) participated in a web-based survey and reported an anger problem before taking AAS. Four measurements of anger were analyzed before, during, and after AAS use. *Anger intensity* was ranked as: 1 = Mild, 2 = ModMild, 3 = Moderate, 4 = ModSev, 5 = Severe; *anger control* was ranked as 1 = Poor, 2 = PoorMod, 3 = Moderate, 4 = ModGood, 5 = Good; *anger frequency* was ranked as 1 = Very rarely, 2 = Rarely, 3 = Occasionally, 4 = Frequently, 5 = Very frequently; *anger duration* was ranked as 1 = Short periods, 2 = Short/Mod, 3 = Moderate, 4 = ModLong, 5 = Long periods.

**Results**

A Friedman test and post-hoc Wilcoxon signed ranks tests with Bonferroni corrections were used to analyze anger intensity, control, frequency, and duration before, during, and after AAS use. *Anger intensity* increased from “mild-moderate” (mean = 1.94) to “moderate” (mean = 2.39) (p < 0.001) from before AAS use to during use and decreased back to “mild-moderate” (mean = 1.68) (p < 0.001) after use. *Anger control* remained in the “poor control” category (mean = 1.81) although improved towards “poor-moderate” (mean = 1.90) (p < 0.001) from before AAS use to during use and improved more to “poor-moderate” (mean = 2.28) (p < 0.001) after use. *Anger frequency* remained in the “rarely” category (mean = 2.08) but increased towards “occasionally” (mean = 2.16) (p < 0.001) from before AAS use to during use and improved to “very rarely” (mean = 1.75) (p < 0.001) after use. *Anger duration* remained in the “short-mod periods” (mean = 2.09) category but improved towards “short periods” (mean = 2.05) (p = 0.001) from before AAS use to during use and improved more to “short periods” (mean = 1.86) (p < 0.001) after use.

**Conclusion**

NMAAS users with pre-existing anger issues reported increased anger intensity and frequency during use. However, they also endorsed better anger control and shorter anger duration during AAS use. Upon stopping AAS, participants reported improvements in all four anger measurements as compared to pre-AAS levels.

**Acknowledgments**

The authors acknowledge the data collection of the original research team of “A league of their own: demographics, motivations and patterns of use of 1,955 male adult non-medical anabolic steroid users in the United States,” www.ncbi.nlm.nih.gov/pmc/articles/PMC2131752/

## P32 Sex-dependent BCAA metabolism as a function of training background and exercise modality

### Joseph K. Pellegrino, Christopher E. Ordway, Marissa L. Bello, Alan J. Walker, Sean P. Conway, Peter J. Gillies, Shawn M. Arent

#### IFNH Center for Human Health and Performance, Rutgers University, New Brunswick, NJ, 08901, USA

##### **Correspondence:** Joseph K. Pellegrino (JoePell@rutgers.edu)

**Background**

Though the branched-chain amino acids (BCAA’s) play a primary structural role, their secondary metabolic role is also important. BCAA’s account for 20-25% of dietary protein, yet bypass splanchnic oxidation, making the major site of BCAA use skeletal muscle. Serum concentrations of BCAA’s and downstream metabolites during and after exercise may provide insight into the metabolic flux and dietary needs associated with exercise.

**Materials and Methods**

Four groups of 10 individuals grouped by sex (M/F) and training history (endurance=END, Resistance=RES; %BF_M-END_=10.1±4.1%; %BF_F-END_=23.3±5.3%; %BF_M-RES_=15.5±6%; %BF_F-RES_=23.4±4.5%; VO_2_max_M-END_=57.5 ±7.5 ml/kg/min; VO_2_max_F-END_=44.9±3.1 ml/kg/min; VO_2_max_M-RES_=41.5±3.7 ml/kg/min; VO_2_max_F-RES_=41.8±2.5 ml/min/kg) underwent 45-min bouts of each aerobic-cycling (AE) or anaerobic weight-training (WT) separated by 3-7 days. Serum was collected before, 0, and 60 minutes post-exercise, and analyzed for AA and downstream metabolite concentrations via UHPLC/MS by Metabolon. RM-ANOVA’s were conducted on log-transformed-median-scaled outputs with significance set at P<0.05. Heat-maps and fold-of changes were generated from the metabolomics response.

**Results.**

BCAA levels declined throughout the exercise & recovery period for all group & exercise combinations. Two sex-specific interactions were revealed regarding the time-course of BCAA metabolism with exercise. Across training background, females showed 2 patterns: a decrease in [BCAA] during WT, which was maintained throughout recovery (pre-post_0_= 0.83±0.04 and pre-post_60_= 0.79±0.06, P<0.05; post_0_-post_60_= 0.94±0.03, P>0.05); no change during AE with a significant decline post-exercise (pre-post_0_= 0.98±0.03, P>0.05; pre-post_60_= 0.86±0.03 and post_0_-post_60_= 0.88±0.04, P<0.05). Alternatively, males responded primarily as a function of training background rather than exercise mode. M-RES [BCAA] decreased pre-post_0_ exercise, and stayed below baseline after (pre-post_0_= 0.89±0.02, and pre-post_60_= 0.80±0.04, P<0.05; post_0_-post_60_= 0.89±0.04, P>0.05), while M-END experienced no change during, followed by a post-exercise drop in [BCAA] (pre-post_0_= 0.93±0.02, P>0.05; pre-post_60_= 0.81±0.06 and post_0_-post_60_= 0.87±0.05, P<0.05). Immediate 2-methyl-oxo-BC-keto-acid products significantly increased during exercise and returned to baseline during recovery, whereas more downstream catabolites increased during exercise, and remained significantly elevated during recovery for all groups and sessions (BCKA_AVG_: pre-post_0_=1.77±0.34, P<0.05, pre-post_60_= 1.06±0.21, P>0.05; post_0_-post_60_= 0.63±0.15, P<0.05; Downstream-Catabolite_AVG_: pre-post_0_= 1.46±0.35 and pre-post_60_= 1.29±0.26, P<0.05; post_0_-post_60_= 0.90±0.13, P>0.05).

**Conclusions.**

The time-course of BCAA metabolism in response to exercise exhibited differential patterns: mode of exercise was the primary stimulus for females and training-background was the distinguishing factor for males. M-RES and F doing WT tended to utilize BCAA during exercise, while there was a more delayed metabolism for M-END and F following AE. These findings are in accord with data describing utilization of multiple substrates during and after exercise in differentially-trained males and females.

**Acknowledgements**

Funding provided by the NJ Institute for Food, Nutrition & Health.

## P33 The effect of training background and sex on the cytokine response to exercise

### Christopher E. Ordway, Joseph K. Pellegrino, Marissa L. Bello, Alan J. Walker, Sean P. Conway, Anthony N. Poyssick, Peter J. Gillies, Shawn M. Arent

#### IFNH, Center for Health and Human Performance, Rutgers University, New Brunswick, NJ, 08901, USA

**Background**

Exercise-related cytokine release is central to both inflammatory response management and substrate utilization. This study examined the effect of training background and sex on the cytokine response from either aerobic or anaerobic exercise bouts.

**Materials and Methods**

Participants (N=40) consisted of endurance (END, n=20) and resistance (RES, n=20) trained individuals, with each group comprised of 10 males and 10 females (AGE_M-END_=24.8±4.9 AGE_F-END_=21.9±2.4 AGE_M-RES_=23.2±2.8 AGE_F-RES_22.3±2.1 yrs; %BF_M-END_=10.1±4.1%BF_F-END_=23.3±5.3%BF_M-RES_=15.5±6%BF_F-RES_23.4±4.5%; VO_2_max_M-END_=57.5 ±7.5 VO_2_max_F-END_=44.9±3.1 VO_2_max_M-RES_=41.5±3.7 VO_2_max_F-RES_41.8±2.5 ml/min/Kg). Using a within-subjects design, participants completed a 45-minute aerobic cycling session (AE) or a 45-minute lower body resistance session (WT) on separate days. Serum was collected before, 0, and 60 minutes post-exercise (T_0_, T_1_, T_2_, respectively), and analyzed for IL-6, IL-10, and growth hormone (GH). A 2x2x2x3 RM MANOVA was run with univariate follow-ups. Significance was set at P<0.05.

**Results**

IL-6 showed trends for an increase from T_0-1_ with a return towards baseline from T_1-2_ (T_0_=12.93±15.55; T_1_=15.36±17.15; T_2_=13.99±15.45 ng/ml, P_T0-1_<0.1, P_T1-2_<0.1). This pattern was most apparent in RES-males. Moreover, END had significantly higher resting IL-6 than RES (END=17.45±17.34, RES=8.41±12.14 ng/ml, P<0.05), and males had a trend for higher values than females (M=16.08±19.24, F=9.79±9.97 ng/ml, P<0.1). IL-10 had a significant main effect for time (P<.05) and a trend for a sex by condition interaction (P<0.1). There was a significant increase from T_0-1_ and IL-10 remained above baseline at T_2_ (T_0_=15.37±23.31 vs T_1_=26.14±39.47 & T_2_=25.87±28.49 ng/ml, P<0.05). The females had a blunted IL-10 response, with no difference between conditions. In the males, greater IL-10 secretion was seen overall, with AE appearing to produce a pronounced response. Pairwise comparisons displayed a significant rise in GH from T_0-1_ with a return to baseline by T_2_ (T_0_=4.87±6.44; T_1_=15.01±15.23; T_2_=4.12±3.61 ng/ml, P_T0-1_<0.05, P_T1-2_<0.05, P_T0-2_>0.05). Area under the curve analysis showed a consistently greater GH response for males than females, and for a strong influence of training background (RES greater) and exercise mode (AE greater) over the total 2-hour period.

**Conclusion**

There was notable individual variability in the cytokine responses. However, sex differences persisted, with males having larger fluctuations from baseline for IL-6, IL-10, and GH. The IL-6 response was robust across conditions, and there was evidence of chronic elevation of this system within the male-END subset. IL-10 variability was apparent, but condition appeared more impactful on secretion in the males. Lastly, GH response displayed sensitivity to exercise type (AE > WT) and training background (RES > END), with male-RES showing the largest responses.

**Acknowledgements**

Funding provided by the NJ institute for Food, Nutrition & Health.

## P34 The body composition effects of extra protein in elite mixed martial artists undergoing frequent training over a six-week period

### Douglas Kalman^1,2^, Alison Escalante^3^

#### ^1^Nutrition Research Unit. QPS. Miami, FL. USA; ^2^Athletics Department, Florida International University, Miami, FL. USA; ^3^Nutrition and Dietetics, Florida International University, Miami, FL. USA

##### **Correspondence:** Douglas Kalman (douglas.kalman@qps.com)

**Background**

Elite athletes often undergo high volume and or intensity training. This volume may affect ability to gain or maintain muscle as well as effect the immune system.

**Materials and Methods**

Eleven healthy men (age 28.1±2.63 yrs, convenience sample), entered this prospective pilot trial, and after signing an informed consent underwent anthropometric and baseline body composition testing. Participants were divided to receive Whey Protein or Rice Protein (NutraBio Whey Isolate/Growing Naturals Rice Protein Isolate) and instructed to supplement with 3 scoops per day (75 grams Pro), with at least 1 scoop (25 gmPro) being ingested after the first training session of the day. Both proteins were tested for banned substances for sport (BSCG, Los Angeles, CA). Subjects engaged in MMA training under the supervision of their coaches (Combat Club, Lantana, FL.) standardized for two sessions per day for five days per week with one session per weekend plus two strength and conditioning sessions per week. Body composition was measured using the BodyMetrix Pro Ultrasound (IntelaMetrix, Brentwood, CA.). All pa:rticipants were asked to maintain their typical diet.

**Results**

The Whey and Rice groups were not different at baseline with respect to body weight (p=0.12), body mass index (p=0.49), percent body fat (p=0.95), fat-free mass (p=0.13), and fat mass (p=0.49). Over the six weeks, within group changes for the Whey group included non-significant changes in body weight (193±31.5 lbs to 195±34.3 lbs; p=0.398), % body fat (11.1±4.33% to 11.3±2.89% p=0.868), FFM (171±26.7 lbs to 173±27.5 lbs; p=0.617) and FM (21.7±9.85 lbs to 22.5 ±8.35 lbs;p=0.782). In these same six weeks, the Rice group (n=5) experienced changes: weight (167±13.6 lbs to 164±12.5 lbs;p=0.159), % body fat (10.9±3.02% to 7.62±1.76%;p=0.098), FFM (149±13.6 lbs to 152±13.2 lbs;p=0.090), and FM (18.2±5.12 lbs to 12.5±2.4 lbs;p=0.095). In terms of between group observations at week six, body weight was not different (p=0.1443), % body fat trended for difference for the Rice group (p=0.05486), FFM was not different between groups (p=0.317) and FM was also not significantly different between the groups (p=0.5486).

**Conclusions**

Adding a mean of 0.92 gm/kg BW Protein (0.41 gm/lb BW) to the diet of an elite level mixed martial artist appears to support maintenance of body composition and fat-free mass while undergoing high volume and intensity training. It does not appear that there was any benefit to one protein source over another, both Whey and Rice protein results were statistically similar. More research is warrented.

**Disclosure**

This study was funded by a research grant from Growing Naturals to Combat Club (Lantana, Florida). The authors consult for Combat Club and have no conflicts of interest to disclose.

## P35 The effects of Teacrine and caffeine on endurance and cognitive performance during a simulated match in high-level soccer players

### Marissa L. Bello, Alan J. Walker, Bridget A. McFadden, David J. Sanders, Shawn M. Arent

#### IFNH Center for Health and Human Performance, Rutgers University, New Brunswick, NJ, 08901, USA

**Background**

Theacrine (1,3,7,9-tetramethyluric-acid) is a pure alkaloid with a similar structure to caffeine and acts comparably as an adenosine receptor antagonist. Early studies have shown non-habituating effects, including increases in energy, focus, and concentration in Teacrine®, the compound containing pure theacrine. The purpose of this study was to determine and compare the effects of Teacrine® and caffeine on cognitive performance and time-to-exhaustion during a simulated soccer game in high-level male and female athletes.

**Materials and Methods**

Participants (N=24; M_Age_=20.96±2.05y, M_MaleVO2max_=55.31±3.39 mL/O_2_/kg, M_FemaleVO2max_=50.97±3.90 mL/O_2_/kg) completed a simulated 90-min soccer match protocol on a treadmill, with cognitive testing including simple reaction time (SRT); choice-RT during a go/no-go task; and complex-RT during a dual task of go/no go with distraction math questions at half-, and post-game. Post-game testing was followed by a run to exhaustion at 85% VO_2max_. Participants completed four sessions in randomized order consisting of ingestion of either 275 mg teacrine (TCr), 275 mg caffeine (Caf), 125/150 mg teacrine+caffeine (TCr+Caf), or placebo(P) 30 min prior to the match. Time of day and pre-exercise nutrition was controlled. RM-MANOVAs with univariate follow-ups were conducted and significance was set at P<0.05.

**Results**

Time-to-exhaustion trended toward improvements in all conditions when compared to placebo (ES_TCr_=0.43, ES_Caf_=0.41, ES_TCr+Caf_=0.51). There was a condition main effect (P<0.05) in which Caf (0.60±0.011 s) and TCr+Caf (0.59±0.012 s) improved choice-RT compared to P (0.61±0.013 s). There was a significant Time main effect for complex-RT errors, with improved accuracy at post compared to mid (16.46±2.02 vs. 19.20±2.13). A Time main effect also occurred for SRT, with better RT at mid compared to post (0.64±0.011 s vs. 0.65±0.011 s). However, a Time x Condition interaction (P<0.05) revealed that P improved from mid to post instead (0.65±0.012 s vs. 0.63±0.010 s).

**Conclusion**

The 27-38% improvements in time-to-exhaustion reflect an increased performance capacity with these supplements that may have important implications for “added time” scenarios. The larger improvement in choice-RT from TCr+Caf may be due to overlapping peak times for the supplements, leading athletes to sustain greater focus under fatigue for longer periods compared to the other conditions. Peak times may also play a role as the largest SRT improvements occurred at mid compared to post-game; perhaps a higher dosage would cause less of a decline during the transition between Caf and TCr. The improvement seen in accuracy post-game may indicate a training effect for allocation of resources toward the end of a game when players need greater concentration.

**Acknowledgments**

Funding provided by Compound Solutions Inc.

## P36 Effects of a single dose of TeaCrine®, caffeine, or their combination on subjective feelings, cognitive performance, and hemodynamics in men and women

### Matthew Butawan, Michelle B. Stockton, Richard J. Bloomer

#### The University of Memphis, Center for Nutraceutical and Dietary Supplement Research, School of Health Studies, Memphis, TN, USA

##### **Correspondence:** Richard J. Bloomer (rbloomer@memphis.edu)

**Background**

Theacrine is a relatively new dietary ingredient, structurally similar to caffeine, and reported to improve mood and cognition in human subjects. The primary purpose of the present study was to determine the tolerability and safety effects of theacrine (i.e., heart rate [HR] and blood pressure [BP]), as well as the impact of theacrine on subjective feelings and cognitive performance in men and women.

**Methods**

24 men (aged: 24.3±6.1) and 26 women (aged: 23.4±3.5) ingested a placebo, theacrine (Teacrine™, Compound Solutions, Inc.) at 25 mg, at 125 mg, caffeine at 150 mg, or theacrine at 125 mg + caffeine at 150 mg on five separate occasions, separated by approximately one week. HR and BP were measured before ingestion and at 30 minutes, 1, 2, 3, 4, and 5 hours post ingestion. Subjects also rated their subjective feelings using a 10 cm visual analog scale at the above times, and performed the trail making test (TMT) of cognitive performance at baseline and at hours 2 and 4 post ingestion. Within conditions changes were assessed with Bonferonni adjustments for pairwise comparisons with significance set at p ≤ 0.05.

**Results**

All 50 subjects successfully completed the study without any adverse eventTGhiss or other issues associated with ingestion of the treatments. Subjective feelings of attentiveness, focus, and energy improved with all active treatments. Grogginess and lethargy significantly declined over time with all treatments. More favorable scores were generally associated with the caffeine and theacrine+caffeine treatments. Caffeine and theacrine+caffeine resulted in a significant increase in subjective focus from baseline to 2 hours post-ingestion, while the 125 mg theacrine treatment reached statistical significance at 3 hours post-ingestion similar results were not observed in the placebo condition. Sense of energy significantly increased from baseline to 2 hours post-ingestion in caffeine and theacrine+caffeine treatments; meanwhile, 125 mg theacrine produced a significant rise in energy 3 hours post-ingestion. Treatment with theacrine had little impact on HR and BP, with marginal increases (~3 bpm; ~3 mm Hg) and no condition by time interactions noted (p>0.05). No condition effects were noted for the TMT (p>0.05), although a trend was present (p=0.069) for theacrine+caffeine, with TMT time improved at 4 hours post ingestion versus pre.

**Conclusions**

These findings indicate that theacrine, when used alone at 125 mg or in combination with caffeine, is safe and effective at improving subjective feelings related to energy in healthy men and women without significantly affecting HR or BP. Theacrine+caffeine may moderately improve cognitive performance as assessed by the TMT.

**Acknowledgements**

Funding for this work was provided by Compound Solutions, Inc. MB and MBS have no conflicts of interest to disclose. RJB has received research funding from and acted as a consultant to dietary supplement companies.

## P37 Resistance training combined with diet decreases body fat while preserving lean mass independent of resting metabolic rate

### Stephanie Mull^1^, Brad Schoenfeld^2^, James Krieger ^3^, Todd Miller^1^

#### ^1^Milken School of Public Health, George Washington University, Washington, D.C., USA; ^2^Department of Health Sciences, CUNY Lehman College, Bronx, NY, USA; ^3^Weightology, LLC, Issaquah, WA, USA

##### **Correspondence:** Stephanie Mull (smull@gwu.edu)

**Background**

The purpose of this study was threefold: (1) To determine whether RT plus dietary intervention (RT+DIET) results in greater improvements in body composition compared with RT or DIET alone; (2) To determine whether RT plus dietary intervention (RT+DIET) results in greater improvements in fat mass in the visceral depot compared with RT or DIET alone, and; (3) To determine whether concomitant increases in muscle mass and decreases in fat mass can occur while in a caloric deficit.

**Methods**

Subjects were 40 obese, premenopausal female volunteers (Body mass = 87.4±12.6; Height = 165.7±7; Age = 32.3±4.8; BMI = 31.9±4.4), randomly assigned to one of four groups: Resistance Training only (RT n=10); Dietary intervention only (DIET n=10); Resistance Training plus Diet (RT+DIET n=10); Control (CON n=10). Baseline measures of body composition were obtained via Dual Energy X-ray Absorptiometry (DXA) and for resting metabolic rate (RMR) via indirect calorimetry. Follow-up DXA scans were obtained at weeks 4, 8, 12 and 16 and RMR testing was repeated at week 16. Subjects in DIET and RT+DIET were provided with daily macronutrient and calorie goals based on their DXA and RMR tests by a registered dietitian, with protein maintained at 1.4 g/lb. FFM/day. Subjects in the RT and RT+DIET groups performed a supervised RT program consisting of exercises for all the major muscle groups. Subjects trained 3 times per week for weeks 1-3 of each month, then trained twice weekly during the 4^th^ week of each month.

**Results**

There was a significant month by group interaction for fat mass. There was no significant linear trend for control. The treatment groups all showed significant linear decreases in fat mass, but the slope of the decrease became progressively steeper from the resistance-training only group, to the diet-only group, to the resistance-training+diet group. There was a significant linear increase for lean mass in resistance training-only. There was no significant month by group interaction or group effect for RMR.

**Conclusions**

Significant reductions in fat mass were achieved by all experimental groups, but results were maximized by a combination of RT and diet. Only the RT group showed significant increases in lean mass. RMR remained unchanged over the course of the study period.

**Acknowledgements**

Funded by the Redstone Global Center for Prevention and Wellness.

## P38 Impact of Beta-alanine supplementation on blood lactate changes and lower body power in collegiate rugby athletes

### C. Smith^1^, R. Stecker^1^, P. Harty^1^, B. Gieske^1^, M. Altepeter^1^, K. Tobey^1^, J. Mike^1^, C. Schroeder^1^, T. VanDusseldorp^2^, K. Escobar^3^, C. Kerksick^1^

#### ^1^Lindenwood University, St. Charles, MO USA; ^2^Kennesaw State University, Kennesaw, GA USA; ^3^University of New Mexico, Albuquerque, NM USA

##### **Correspondence:** C. Kerksick (ckerksick@lindenwood.edu)

**BACKGROUND**

Beta-alanine (BA) is a precursor to carnosine, a key intracellular buffer that assists in the maintenance of intracellular pH during high-intensity efforts. Rugby consists of multiple intermittent periods of maximal or near-maximal efforts with short periods of rest/active recovery. This study aimed to evaluate the impact of 6wks of BA supplementation on anaerobic performance measures in collegiate rugby players.

**METHODS**

Sixteen male, collegiate rugby players (21±1.5 yrs; 179±6.2 cm; 91.2±11.1 kg; 20.1±4.3% body fat) were randomized in a double-blind, placebo controlled manner to consume 6.4 g/d of placebo (PLA) or BA. In identical pre/post testing sessions, anaerobic endurance and strength endurance was assessed. Anaerobic endurance was assessed using distance covered during a field-based intermittent sprint running test with blood lactate (BLa) collections at rest, after the 2^nd^ and 4^th^ sets, and immediately post completion. During a five set to fatigue protocol at 70%1RM examining lower body (back squat) strength endurance, lower body peak power (LPE) and average power (LAE) endurance were evaluated by changes in peak power and average power between all sets. Data was analyzed using 2x2 (group x time) mixed factorial ANOVAs with repeated measures on time. Data is presented as means ± SD.

**RESULTS**

Pre/post resting BLa levels were not different (p>0.05) between groups, but resting BLa levels did decrease (p<0.05) in both groups at POST vs PRE. BLa levels sharply increased before and after supplementation, with no interaction effect being observed between groups. However, BLa levels in BA were more favorably maintained when compared to PLA from start to completion of running (p < 0.05). Peak and average power values at PRE and POST were similar baseline between groups (p > 0.05). When compared to PRE, peak power values produced throughout the SE protocol decreased similarly in both groups, while average power values did not change. The BA group experienced a significant decrease in peak power from sets 3 to 4 compared to PLA (p = 0.03), however no other significance was observed between the other sets for either group. No significant differences were observed in average power for either group.

**CONCLUSIONS**

BA non-significantly attenuated the overall increase in BLa. BA supplementation exerted minimal influence on the maintenance of power in a multi-set, fatiguing resistance exercise. Overall, these results suggest BA likely imparts minimal influence on the maintenance or improvement of anaerobic performance within the parameters of our study design.

**ACKNOWLEDGEMENTS**

The authors thank CarnoSyn for product donation and blinding and the NSCA Foundation for funding this project and the Lindenwood University Men’s Rugby team and coaches for their participation.

## P39 The effects of whey versus casein protein supplementation on body composition and resting metabolic rate

### Corey A. Peacock^1^, Kristain Mejia^1^, Tobin Silver^1^, Gabriel J. Sanders^2^, Jose Antonio^1^

#### ^1^Department of Health and Human Performance, Nova Southeastern University, Davie FL USA; ^2^Department of Kinesiology, Northern Kentucky University, Highland Heights KY USA

##### **Correspondence:** Jose Antonio (ja839@nova.edu)

**Background**

Prior research has shown that protein supplementation may have a positive effect on body composition; however, minimal research exists comparing whey to casein protein. Therefore, the purpose of the current study is to compare whey versus casein in regards to body composition and resting energy expenditure.

**Methods**

Eighteen physically trained, healthy individuals (23±3.1 yrs.; 171.7 cm; 12 males; 6 females) completed a randomized two condition [Combat 100% Isolate (Whey) versus Combat 100% (Casein)] by two-time point [Pre-, Post-] intervention. The intervention consisted of subjects supplementing with either whey or casein protein (60 g daily) in conjunction with resistance training over an 8-week period. Body composition was assessed via the the Bod Pod® and resting metabolic rate (RMR) was measured via a Parvo Metabolic Cart (Parvomedics Inc., Sandy, UT) both pre- and post-intervention.

**Results**

There were no differences within or between groups for any of the measures (Table 10).

**Conclusion**

Based on this pilot study, neither protein produced significant changes in body composition or REE over an 8-week treatment period. A study utilizing a larger sample coupled with an aggressive training program would be warranted.

**Acknowledgements**

This study was funded with a grant from MusclePharm®.

**Table 10 (abstract P39).** Body Composition and REE

	WEIGHT PRE (kg)	WEIGHT POST (kg)	BF% PRE	BF% POST	REE PRE (kcal)	REE POST (kcal)
Whey:	81.1±9.2	81.7±8.8	15.4±7.0	15.2±7.2	1994±240	1956±241
Casein:	73.5±12.9	74.5±16.8	20.4±10.9	20.9±11.0	1600±249	1626±218

## P40 Acute effect of citrulline malate supplementation on upper-body resistance exercise performance in resistance-trained men

### Adam M. Gonzalez^1^, Robert W. Spitz^1^, Gerald T. Mangine^2^

#### ^1^Department of Health Professions, Hofstra University, Hempstead, NY, USA; ^2^Department of Exercise Science and Sport Management, Kennesaw State University, Kennesaw, GA, USA

##### **Correspondence:** Adam M. Gonzalez (Adam.Gonzalez@hofstra.edu)

**Background**

The purpose of this study was to examine the effect of supplementation with 8 grams of citrulline malate on acute multi-joint resistance exercise performance, subjective measures of focus, energy, and fatigue, and the muscle swelling response to training in resistance-trained men.

**Materials and methods**

Twelve recreationally resistance-trained men (21.4 ± 1.6 y; 163.0 ± 46.2 cm; 85.0 ± 12.4 kg; 3.5 ± 1.6 y of resistance training experience) underwent two testing sessions administered in a randomized and double blind fashion. During each visit, participants were provided either 8 grams citrulline malate (CM) or a placebo (PL) 40-min prior to beginning a standardized warm-up and initiating a barbell bench press resistance exercise protocol. The resistance exercise protocol consisted of 5 sets of 15 repetitions at 75% 1RM with 2-minute rest intervals. Participants were instructed to complete all 15 repetitions or as many as possible without assistance. The total number of repetitions performed for each set was recorded. Additionally, the average and peak power of each repetition was recorded via an accelerometer. Participants were also given subjective questionnaires upon arrival (BL), 40-min following supplement ingestion (PRE), and immediately after the resistance exercise protocol (IP) to assess feelings of energy, focus, and fatigue, along with perceived exertion. A researcher also assessed muscle thickness of the triceps brachii via ultrasonography at BL, PRE, and IP. The two experimental trials occurred at the same time of day and were separated by approximately one week.

**Results**

Significant (p<0.05) main effects for time were observed for all variables except for subjective feelings of energy (p=0.085). A group × time interaction (F=2.86, p=0.034, n^2^=0.21) was observed for repetitions performed, where participants performed more (p=0.015) repetitions on set 3 during PL (5.7±1.2 repetitions) compared to CM (4.6±1.2 repetitions). However, during set 4, participants tended (p=0.089) to perform more repetitions during CM (4.8 ± 1.8 repetitions) compared to PL (4.3 ± 1.3 repetitions). No other differences were observed between trials.

**Conclusions**

In conclusion, supplementation with 8 grams of citrulline malate 40-mins prior to a low volume barbell bench press resistance exercise protocol did not appear to increase exercise performance, alter subjective measures of focus, energy, and fatigue, or augment the muscle swelling response to training in resistance-trained men.

## P41 Comparison of different RMR prediction equations over a nine-week detraining period in division I female soccer athletes

### K. Levers, T. M. Purdom, D. Wetzel, J. Giles, L. Brown, N. Fry

#### Department of Health, Athletic Training, Recreation and Kinesiology, Longwood University, Farmville, VA 23901, USA

**Background**

Estimating resting metabolic rate (RMR) is a common practice for nutrition professionals. Currently, RMR estimation equations (RMREE) prioritize different variables to predict caloric expenditure which include: height, weight, age, gender, and fat free mass (FFM) which can cause variance in RMREEs. Additionally, periodized changes in training density experienced by Division I athletes cause variation in both body composition and RMR [1,2,3]. However, it is currently unknown how detraining impacts different RMREEs. Therefore, the purpose of this study is to evaluate the variance of multiple RMREEs over a detraining period.

**Materials and Methods**

Caloric expenditure was estimated using five equations: Harris-Benedict (HB), Revised HB, Owen, Mifflin-St. Jeor (MSJ), and Cunningham in 21 female Division I collegiate soccer athletes (19.2 ± 1.04 yrs, 165.3 ± 6.5 cm, 63.4 ± 7.4 kg, 49.8 ± 5.1 kg FFM, 22.2 ± 3.8% body fat). Each subject was tested after the competitive season (B1), and after nine weeks of detraining (B2). Height and weight were measured and body density was calculated via a three-site skinfold with the Brozek formula to estimate body composition. One-way ANOVAs were used to analyze differences between RMREEs within B1 and B2. T-tests compared individual RMREEs across blocks. Each RMR is reported as kilocalories per day (kcal/d)**.** All values are reported as mean ± SD.

**Results**

Statistical analysis revealed that within B1 nearly all the RMRs were significantly different from each other (*p* < 0.001), except for the MSJ and Owen estimations (*p* = 0.118). All RMRs including the MSJ and Owen predictions were significantly different from each other in B2 (*p* < 0.01). Paired t-tests revealed no significant differences (*p* > 0.05) for individual equation comparisons across blocks for all RMREEs.

**Conclusions**

Results indicate that the RMREEs were all significantly different, except for the MSJ and Owen estimations. The literature suggests the need for multiple equations that are population specific due to the prioritization of various anthropometrics to predict RMR [4,5]. The MSJ, Owen, and HB prioritize body weight to calculate RMR and are intended for obese and non-obese populations. However, Burke and Deakin [5] suggest the Cunningham for athletes as FFM is the primary variable. Body composition was not a relevant factor in our analysis, however there is a variance of 207 kcal across RMREEs. Therefore, it is imperative to review the literature to adequately evaluate the accuracy of RMREEs due to the prioritization of various anthropometrics within each equation.

1. Drenowatz, C. Hand, G. Shook, R. Jakicic, J. Hebert, J. Burgess, S. Blair, S. The association between different types of exercise and energy expenditure in young nonoverweight and overweight adults. *Applied Physiology, Nutrition, and metabolism*. 2015. 40(3). 211-217.

2. Silva, JR. Brito, J. Akenhead, R. Nassis, GP. The transition period in soccer: a window of opportunity. *Sports Medicine*. 2016. 46(3). 305-313.

3. Westerterp, KR. Alterations in energy balance with exercise. *American Journal of Clinical Nutrition*. 1998. 68(4). 970S-974S.

4. Freakenfield, D. Roth-Yousey, L. Compher, C. Comparison of predictive equations for resting metabolic rate in healthy nonobese and obese adults: A systematic review. *Journal of the American Dietetic Association*. 2005. 105. 775-789.

5. Burke, L. and Deakin, V. Energy requirements of the athlete: assessment and evidence of energy efficiency. In *Clinical Sports Nutrition*. 5^th^ Ed. 2015. McGraw-Hill Education. 120-127.

## P42 A natural nootropic spearmint extract is safe and well-tolerated in young, healthy individuals

### Kelli Herrlinger^1^, Brandon Lewis^1^, Aaron Tribby^2^, Jesse Gwinn^2^, Chantelle Slayton^2^, Micah Henigman^2^, Israel Santiesteban^2^, Sarah Dawes^2^, Joanne Lasrado^1^, Brenda Fonseca^1^, Paul Falcone^2^

#### ^1^Kemin Foods, L.C., Des Moines, IA, USA; ^2^MusclePharm Sports Science Institute, Denver, CO, USA

##### **Correspondence:** Kelli Herrlinger (Kelli.Herrlinger@kemin.com)

**Background**

Neumentix™ Phenolic Complex K110-42 (NEU; Kemin Foods, L.C., Des Moines, IA), a natural spearmint extract containing 14.5% rosmarinic acid and 24% total phenolic content with cognitive benefits, has been previously demonstrated to be safe and well-tolerated when administered at 900 mg/day for 90 days to healthy, older adults. The present study builds on the current safety and tolerability data for this natural nootropic in young, healthy adults.

**Methods**

Recreationally-active men and women (n=142; NEU: 27.2 ± 0.9 y; placebo [PLA]: 27.9 ± 0.9 y) were randomized to consume either 900 mg of NEU or a visually-identical placebo (PLA) for 90 days. Safety and tolerance were assessed through blood safety panels, vital signs, inflammatory biomarkers, and monitoring of Adverse Events (AEs). Safety panels included: complete blood count, complete metabolic profile, and blood lipids. Vital signs were measured to investigate changes in hemodynamics. Inflammation was measured by C-reactive protein (CRP) and interleukin-6 (IL-6). Measurements were taken at baseline and at 7, 30, and 90 days during supplementation. AEs were assessed throughout the study.

**Results**

No statistically significant treatment effects were observed in the blood safety panels. Treatment x visit interactions were observed for absolute monocytes and granulocytes; pairwise comparisons revealed significant differences at Day 30, which were no longer evident at 90 days. All values in the blood safety panels were within the accepted physiological ranges. No statistically significant treatment effects were observed for any vital sign; however, a treatment x visit interaction (p=0.029) was observed for systolic blood pressure, resulting from a decrease in PLA. All vital signs remained within the accepted physiological ranges. Raw values for all measures of inflammation decreased over the 90 days in NEU, although no significant between group differences were observed. A trend was observed in treatment x visit interaction for CRP; pairwise comparison revealed significant between group differences at Day 30 (NEU, 1.39 ± 0.228 mg/L vs. PLA, 2.63 ± 0.932 mg/L; Mean ±SEM; p=0.040). There was no difference in total number of AEs or total number of AEs related to the study product between groups.

**Conclusion**

The present study confirms that chronic supplementation of 900 mg Neumentix in healthy adults is safe and well-tolerated. The positive trends with CRP warrant follow-up studies given the advantages for reducing inflammation after intense physical activity or sports performance. The current data further builds on the body of evidence for Neumentix as a safe and natural nootropic.

**Acknowledgements**

The present study was funded by Kemin Foods L.C.

## P43 Chronic supplementation with a natural nootropic spearmint extract improves active reaction performance in young, healthy individuals

### Paul Falcone^1^, Aaron Tribby^1^, Jesse Gwinn^1^, Chantelle Slayton^1^, Micah Henigman^1^, Israel Santiesteban^1^, Sarah Dawes^1^, Joanne Lasrado^2^, Brenda Fonseca^2^, Brandon Lewis^2^, Kelli Herrlinger^2^

#### ^1^MusclePharm Sports Science Institute, Denver, CO, USA; ^2^Kemin Foods, L.C., Des Moines, IA, USA

##### **Correspondence:** Kelli Herrlinger (Kelli.Herrlinger@kemin.com)

**Background**

Studies have demonstrated that Neumentix^TM^ Phenolic Complex K110-42 (NEU; Kemin Foods, L.C., Des Moines, IA), a natural spearmint extract containing 14.5% rosmarinic acid and 24% total phenolic content, can improve cognition. Given the growing interest in connecting mental and physical performance, the current study examined whether the nootropic benefits of Neumentix supplementation translate into improvements in active cognitive performance in young, healthy adults.

**Methods**

In a double-blind, placebo-controlled, parallel design, 142 recreationally-active adults (NEU: 27.2±0.9y; placebo [PLA]: 27.9±0.9y) were randomized to consume 900 mg of NEU or a visually-identical PLA for 90 days. Choice reaction performance was measured as hits and average reaction time (ART) on a three-tower testing device (Makoto Arena II; Makoto USA Inc., Elk Grove Village, IL) via six tests: stationary, lateral, and multi-directional, each administered with or without footplates. Measurements were taken at baseline,7, 30, and 90 days during supplementation. Data are shown as mean ±SEM.

**Results**

A treatment effect (p=0.019) was observed for hits on the stationary test with footplates, pairwise comparisons revealed significant differences at Day 30 (NEU: 28.96±0.275 hits vs. PLA: 28.09±0.256 hits; p=0.040) and Day 90 (NEU: 28.42±0.352 hits vs. PLA: 27.02±0.487 hits; p=0.002) compared to placebo. A treatment effect (p=0.036) was observed for ART on the stationary test with footplates and pairwise comparisons revealed significant differences at Day 7 (NEU: 0.5896±0.0074 sec vs. PLA: 0.6141±0.0096 sec; p=0.049) and Day 30 (NEU: 0.5811±0.0090 sec vs. PLA: 0.6033±0.0074 sec; p=0.049) compared to placebo. A treatment effect (p=0.020) was observed for - hits on the multi-directional test with footplates, and pairwise comparisons revealed significant differences at Day 30 (NEU: 19.25±0.244 hits vs. PLA: 18.45±0.198 hits; p=0.007) and Day 90 (NEU: 19.39±0.263 hits vs. PLA: 18.66±0.225 hits; p=0.026) compared to placebo. There were no significant differences observed for the remaining Makoto tests.

**Conclusion**

The present study indicates that 900 mg of Neumentix improves ART in a stationary test of choice reaction performance as early as 7 days and enhances hit rate in both stationary and multi-directional testing following 30 days of supplementation with the effect still present at 90 days in young, healthy individuals. The unique device used was selected because it links cognitive function to active performance. The current data confirm previous work, and provide evidence that the cognitive benefits of Neumentix would be applicable to an athletic context (training, practice, or competition), thus further supporting Neumentix as a safe and natural nootropic.

**Acknowledgements**

The present study was funded by Kemin Foods L.C.

## P44 The effect of the addition of an amylopectin/chromium complex to increasing doses of whey protein on muscle protein synthesis in rats

### James Komorowski^1^, Sara Perez Ojalvo^1^, Nurhan Sahin^2^, Hakki Tastan^3^, Kazim Sahin^2^

#### ^1^Nutrition 21, LLC, Purchase, NY, USA; ^2^Firat University, Elazig, Turkey; ^3^Gazi University, Ankara, Turkey

##### **Correspondence:** James Komorowski (jkomorowski@nutrition21.com)

**Background**

In a previous clinical study, an amylopectin/chromium complex (ACr; Velositol®) was shown to double muscle protein synthesis (MPS) when added to 6 g of whey protein (WP) compared to WP alone. The purpose of this study was to examine the effects of ACr when added to increasing doses of WP to determine if benefits seen in the clinical study could also apply to higher doses of WP.

**Methods**

Young (8-week old) male Wistar rats (250-300 g) were randomized into nine groups (n=8 in each group) (Table 11).

On the day of the single-dose experiment, rats were exercised at 26 m/min for 2 hours and then fed protein or water according to their assigned group. Approximately one hour later, rats were injected with a bolus dose (250 mg/kg body weight, 25 g/L) of labeled phenylalanine, and ten minutes later, muscle tissue samples were taken to measure the fractional rate of protein synthesis (FSR).

**Results**

**Fig. 1 (abstract P44).**
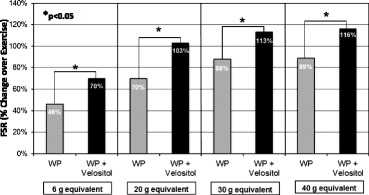
FSR (% Change over Exercise)

In this study, all active treatment groups (II - IX) increased MPS compared to the exercise control group (p<0.05). However, all the WP plus ACr groups (VI, VII, VIII and IX) increased MPS over their corresponding WP alone groups (Fig. 1; p<0.05).

**Conclusions**

Within the confines of this preclinical study design, the addition of ACr to increasing doses of WP enhanced exercise-induced MPS over whey protein alone, providing evidence that the beneficial effects seen in the clinical study using 6 g of whey protein can also occur when using higher doses of whey protein. In addition, the maximum FSR levels seen in the ACr groups were higher than the maximum FSR levels achieved with whey protein alone.

**Acknowledgements**

This study was funded by Nutrition21, LLC.

**Table 11 (abstract P44).** See text for description

	Group	Human Equivalent WP Dose	Human Equivalent ACr Dose
(I)	Exercise Control Group	0 g	0 g
(II)	Exercise plus WP (0.465 g/kg BW)	6 g	0 g
(III)	Exercise plus WP (1.55 g/kg BW)	20 g	0 g
(IV)	Exercise plus WP (2.33 g/kg BW)	30 g	0 g
(V)	Exercise plus WP (3.1 g/kg BW)	40 g	0 g
(VI)	Exercise plus WP (0.465 g/kg BW) and ACr (0.155 g/kg BW)	6 g	2 g
(VII)	Exercise plus WP (1.55 g/kg BW) and ACr (0.155 g/kg BW)	20 g	2 g
(VIII)	Exercise plus WP (2.33 g/kg BW) and ACr (0.155 g/kg BW)	30 g	2 g
(IX)	Exercise plus WP (3.1 g/kg BW) and ACr (0.155 g/kg BW)	40 g	2 g

## P45 Muscle protein synthesis of pea protein is significantly enhanced with the addition of an amylopectin/chromium complex

### James Komorowski^1^, Sara Perez Ojalvo^1^, Cemal Orhan^2^, Kazim Sahin^2^

#### ^1^Nutrition 21, LLC, Purchase, NY, USA; ^2^Firat University, Elazig, Turkey

##### **Correspondence:** James Komorowski (jkomorowski@nutrition21.com)

**Background**

Prior studies have shown that an amylopectin/chromium complex (ACr; Velositol®) significantly enhances the ability of whey protein to increase muscle protein synthesis (MPS). This preclinical study was designed to further examine the effects of ACr when added to a different protein source. This time, a plant-based protein source was selected to determine if benefits seen in the clinical study could also apply to plant proteins. The purpose of this preclinical study was to evaluate if the addition of ACr to pea protein could increase MPS after exercise compared to pea protein and exercise alone. Pea protein contains ~8% leucine by weight compared to ~11% in whey protein.

**Methods**

Male Wistar rats (8-week old) weighing approximately 250-300 g were reared at 22 ± 2 °C in a 12/12 hour light/dark cycle and randomized into three groups (n=8 in each group): (I) Exercise control group; (II) Exercise plus pea protein (0.465 g/kg BW equivalent to a 6 g human dose); (III) Exercise plus pea protein (0.465 g/kg BW equivalent to a 6 g human dose) and ACr (0.155 g/kg BW equivalent to a 2 g human dose).

Rats were acclimated using a 10-day treadmill schedule that gradually increased in speed and duration up to 26 m/min for 15 minutes. On the day of the single-dose experiment, rats were exercised at 26 m/min for 2 hours and then fed protein or water according to their assigned group, immediately after exercise. Study product was dissolved in water and administered by oral gavage. Approximately one hour later, rats were injected with a bolus dose (250 mg/kg body weight, 25 g/L) of phenylalanine labeled with deuterium to measure the fractional rate of protein synthesis (FSR) and ten minutes later, muscle tissue samples were taken to determine MPS measured by FSR.

**Results**

**Fig. 2 (abstract P45).**
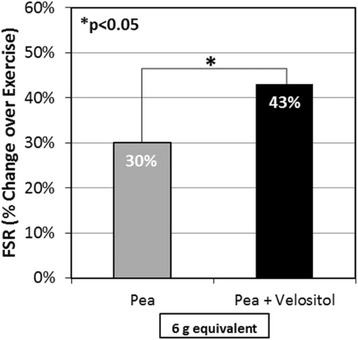
See text for description

Compared to the exercise control group, both treatment groups (II and III) increased MPS (p<0.05). However, the pea protein plus ACr group (III) increased MPS by 43% over the exercise control group, compared to a 30% increase in the pea protein group (II) (Fig. 2; p<0.05 between treatment groups).

**Conclusions**

As demonstrated in this preclinical study, the addition of ACr to pea protein enhanced MPS by 43% over pea protein alone, providing evidence that the beneficial effects seen in the clinical study using whey protein may also occur when using other sources of plant-based (e.g., pea) protein.

**Acknowledgements**

This study was funded by Nutrition21, LLC.

## P46 The effect of the addition of an amylopectin/chromium complex to branched-chain amino acids on muscle protein synthesis in rats

### James Komorowski^1^, Sara Perez Ojalvo^1^, Mehmet Tuzcu^2^, Kazim Sahin^2^

#### ^1^Nutrition 21, LLC, Purchase, NY, USA; ^2^Firat University, Elazig, Turkey

##### **Correspondence:** James Komorowski (jkomorowski@nutrition21.com)

**+Background**

In a previous clinical study, an amylopectin/chromium complex (ACr; Velositol®) was shown to significantly increase muscle protein synthesis (MPS) when added to 6 g of whey protein compared to whey protein alone. In that study, ACr doubled the impact of protein on MPS. The purpose of this preclinical study was to evaluate if the addition of ACr to branched-chain amino acids (BCAA) could increase MPS after exercise compared to BCAA and exercise alone. BCAA consist of a combination of the amino acids leucine, isoleucine, and valine most commonly in a 2:1:1 ratio. BCAA contains 50% leucine content compared to 11% in whey protein.

**Methods**

Young (8-week old) male Wistar rats weighing approximately 250-300 g were reared at 22 ± 2 °C in a 12/12 hour light/dark cycle and randomized into three groups (n=8 in each group):

(I) Exercise control group
(II) Exercise plus BCAA (0.465 g/kg BW equivalent to a 6 g human dose)
(III) Exercise plus BCAA (0.465 g/kg BW equivalent to a 6 g human dose) and ACr (0.155 g/kg BW equivalent to a 2 g human dose)

All rats completed a 10-day treadmill acclimation schedule that gradually increased in speed and duration up to 26 m/min for 15 minutes. On the day of the single-dose experiment, rats were exercised at 26 m/min for 2 hours and then fed BCAAs or water according to their assigned group, immediately after exercise. The study product was dissolved in water and administered by oral gavage. Approximately one hour later, rats were injected with a bolus dose (250 mg/kg body weight, 25 g/L) of phenylalanine labeled with deuterium to measure the fractional rate of protein synthesis (FSR) and ten minutes later, muscle tissue samples were taken to determine MPS measured by FSR.

**Results**

**Fig. 3 (abstract P46).**
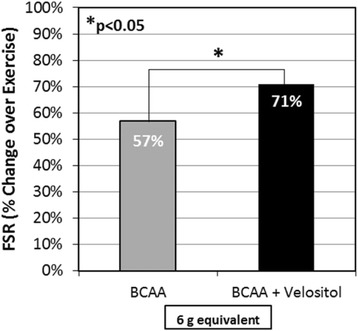
See text for description

Both treatment groups (II and III) increased MPS compared to the exercise control group (p<0.05). However, the BCAA plus ACr group (III) increased MPS by 71% over the exercise control group, compared to a 57% increase in the BCAA group (II) (Fig. 3; p<0.05 between treatment groups).

**Conclusions**

Within the confines of this preclinical study design, the addition of ACr to BCAA enhanced exercise-induced MPS by 25% over BCAA alone, providing evidence that the beneficial effects seen in the clinical study using whey protein, may also occur when using BCAA.

**Acknowledgements**

This study was funded by Nutrition21, LLC.

## P47 Performance and body composition assessment of football players preparing for NFL combine and pro days

### Jaclyn Sklaver^1^, Whitney Hochstetler^2^ Kaylee Williams^3^, Obu Okechukwu Chine Imonugo^3^, Kyle Smith,^2^ Robert Wildman^3^

#### ^1^FitMissNYC, New York, NY, USA; ^2^Institute of Sports Sciences and Medicine (ISSM), Florida State University, Tallahassee FL, USA; ^3^Dymatize Athletic Nutrition Institute (DANI), Dallas TX, USA

##### **Correspondence:** Robert Wildman (rwildman@dymatize.com)

**Background**

Every year hundreds of college football players prepare for the NFL Combine and/or Pro Days. Chip Smith Performance Systems (CSPS) (Norcross, GA) is a major professional/elite training facility and one of the most prominent of these facilities providing a training platform, position-specific coaching and offering chef-prepared meals and nutrition products. The purpose of this investigation is to present anthropometric and performance data from the 2017 Combine Training class at CSPS.

**Materials & Methods**

Fifty-five college players attended Combine/ProDay Training at CSPS for 4-8 weeks, early January to March 2017. Arrival and length of stay were dependent upon end of season/bowl and Combine and/or ProDay schedule. Players were acclimated to on/off-site schedules including field work, strength, power, speed and agility training and position-specific coaching. Most players ate hotel buffet breakfast, received chef-prepared meals for late afternoon/evening and some received nutrition counseling. Nutrition products (Dymatize, Dallas TX; PowerBar, Emeryville CA) including protein (whey, casein), BCAAs, creatine, carbohydrate and fish oil were provided with instructions for use. Training included an emphasis on Combine/ProDay performance measures (e.g. bench press (BP) 225 lb reps to failure), 40-yard dash (40YD), etc. Pre/post body composition (InBody 770, InBodyUSA) and performance data were captured at CSPS or at Combine/ProDay. Players signed an informed consent and study design was simply observational reporting of recorded player data.

**Results**

The number of data points (n) collected for each measure varied. Body weight change varied based player status and position specific goals. Meanwhile, roughly 70% recorded gains in skeletal muscle mass (SMM) (1.1 ± 1.5 kg; 2.3 ± 3.2%) with 58% gaining ≥1 kg and 35% ≥2 kg. Roughly 65% reduced body fat mass (BFM) (-0.4 ± 1.2 kg; -2.3 ± 0.1%) with 35% and 12% reducing fat mass by ≥1 kg and ≥2 kg, respectively. Bench press increased for 95% of players (5.5±2.4 reps) with 71% and 31% increasing ≥5 and 7 reps, respectively. All players assessed got faster in the 40YD (-0.2 ± 0.1 sec).

**Conclusions**

The observational results of this investigation demonstrate that college football players transitioning from end of season/bowl games to Combine/ProDay assessments and training at CSPS with training and nutrition support can make dramatic improvements in body composition and performance measures. These improvements may impact draft position or catalyze undrafted contracts with professional teams. Based on the study design, relationships between specific training and nutrition aspects cannot be made at this time.

**Acknowledgements**

We wish to acknowledge: 1) Chip Smith and coaches at CSPS, 2) InBodyUSA for providing InBody770 BIA, 3) Dymatize and POWERBAR for providing nutrition products.

## P48 Chicken protein isolate and whey protein concentrate comparably elevate resting metabolism

### Courtney M. Nordhus, Jordan M. Joy, Desiree E. Patterson, Roxanne M. Vogel, Thomas H. Hoover, Robert E. C. Wildman, K. Shane Broughton, Nancy M. DiMarco

#### ^1^Department of Nutrition and Food Sciences, Texas Woman’s University, Denton, TX, USA

##### **Correspondence:** Courtney M. Nordhus (cnordhus@twu.edu)

**Background**

Proteins have the greatest thermogenic potential compared to the other macronutrients. Whey protein is a complete protein and considered the standard for supplemental proteins due to its high branched-chain amino acid (BCAA) content. Chicken protein isolate (CPI) is a novel, complete protein with similar concentration of BCAAs. Effects of WPC versus CPI on post-prandial metabolism is unknown. Therefore, this investigation examined acute metabolic responses to isocaloric whey protein concentrate (WPC), carbohydrate (CHO), and CPI supplements.

**Materials and Methods**

In a single-blind design, 26 recreationally active males (174.8±7.5 cm; 77.1±11.1 kg; 22±2 years; 60.3±9.3 kg lean) ingested 0.3 g/kg lean soft tissue WPC (WPC80), CHO (maltodextrin), or CPI (ChikPro, International Dehydrated Foods, Springfield, MO, USA). Resting energy expenditure (REE) and respiratory quotient (RQ) were measured using indirect calorimetry over a 3-hour post-prandial period and compared to a pre-treatment, fasting baseline. Average treatment dose was 18.5 g of protein or CHO. Data were collected at pre, 15, 30, 45, 60, 90, 105, 120, 150, 165, and 180 minutes using the average of the previous 10 data points. Thermogenic effect of food (TEF) was estimated from AUC of the REE delta values from baseline. Data are reported as mean±standard deviation.

**Results**

No differences existed at baseline between treatments for REE or RQ (p>0.05). Delta from baseline indicated significant (p<0.05) elevation of REE for WPC at 60, 90, 105, and 120 minutes post-ingestion (60 m: 0.197±0.107; 90 m: 0.143±0.070; 105: 0.103±0.073; 120 m: 0.107±0.068 Cal/minute) and for CPI beginning at 90 minutes and remaining greater through 180 minutes (90 m: 0.143±0.057; 105: 0.137±0.063; 120: 0.165±0.073; 150: 0.115±0.069; 165: 0.103±0.067; 180: 0.143±0.069 Cal/minute) versus CHO (60 m: 0.019±0.092; 90 m: -0.022±0.056; 105: -0.036±0.071; 120: -0.035±0.085; 150: -0.014±0.085; 165: -0.015±0.075; 180: -0.001±0.059 Cal/minute). TEF was significantly (p<0.05) greater for WPC (+7.9±4.3Cal) than CHO (+3.0±4.5Cal) beginning at 60 minutes post ingestion and for CPI (+10.2±4.5Cal) versus CHO (+2.3±5.9Cal) beginning at 90 minutes post-ingestion and remained greater through 180 minutes (WPC: +20.5±10.4; CHO: +0.6±12.2; CPI: +24.0±9.6Cal).

**Conclusions**

Both WPC and CPI increased REE over 3 hours versus isocaloric CHO. However, CPI has later onset and is sustained for a longer period of time than WPC compared to CHO despite similar total TEF over three hours. Absorption rates and metabolism of WPC and CPI may differ. Metabolic effects of CPI beyond 180 minutes following ingestion and digestion rates between WPC and CPI should be investigated.

**Acknowledgements** – Supported by International Dehydrated Foods (Springfield, MO) and Dymatize Athletic Nutrition Institute (Dallas, TX).

## P49 Changes in urinary indices of hydration and bioimpedance measurements following acute dehydration

### Jordan R. Moon^1,7^, Jordan M. Joy^2^, Eric R. Serrano^3^, Michael P. Kim^4^, Matt M. Mosman^5^, Paul H. Falcone^6^, Chih-Yin Tai^7^, Laura R. Carson^6^, Justen L. Straight^8^, Susie L. Oury^9^, Carlos Mendez Jr. ^10^, Nick J. Loveridge^6^, and Manolis Aivazelis^11^

#### ^1^Impedimed, Inc., Carlsbad, CA, USA; ^2^Texas Woman's University, Denton, TX, USA; ^3^Serrano Family Practice, Columbus, Ohio, USA; ^4^Cedars-Sinai, Los Angeles, California, USA; ^5^NutraBio Labs, Inc., Middlesex, New Jersey, USA; ^6^MusclePharm Corp., Denver, Colorado, USA; ^7^United States Sports Academy, Daphne, Alabama, USA; ^8^Glanbia, Lincoln, Nebraska, USA; ^9^Metropolitan State University of Denver, Denver, Colorado, USA; ^10^University of Colorado Anschutz Medical Campus, Aurora, Colorado, USA; ^11^Impedimed, Inc., Kalamaria, Thessaloniki, Greece

**Background**

Tracking acute changes in hydration is typically performed by measuring changes in body mass. Both bioimpedance devices and urinary indices have been used to determine hydration and fluid volumes and should also detected acute changes in hydration. The purpose of the study was to determine the relationships between urinary and bioimpedance values and changes in body mass after acute dehydration.

**Materials and Methods:**

Nine women and six men (28 +/- 4 yrs, 175 +/- 6.5 cm, 76.7 +/- 11 kg) participated in the study. Pre- and post-testing measurements included nude body mass (BM), urine color (UC), urine specific gravity (USG), single frequency bioimpedance (BIA) variables (R, Xc, Phase) and bioimpedance spectroscopy (BIS) variables (R0, Rinf). Subjects entered the lab in a normally hydrated state (USG: 1.000-1.018) and ran on a treadmill fully clothed at 80% of their predicted max heart rate for 30 minutes followed by sitting in a sauna at 70 degrees Celsius for 15 minute intervals until a reduction in BM by approximately 2% was detected (2.1 +/- 0.3%).

**Results:**

Neither urinary- or bioimpedance-based variables were significantly correlated with the changes in BM (p > 0.05). BIA phase angle was the only variable not significantly different from pre- to post-dehydration (p = 0.146). Urine color increased by 1 (+/- 1.2) with two subjects showing a decrease and three showing no change (p = 0.0059). Urine specific gravity increased by 0.0043 (+/- 0.0044) with one subject showing a decrease and two subjects showing no change (p = 0.002). BIA R values decreased by 19.1 Ohms (+/- 13.5) with no subjects showing an increase (p = 0.00008). BIA Xc values decreased by 3.1 Ohms (+/- 2.2) with two subjects showing an increase (p = 0.00011). BIS R0 values decreased by 28 Ohms (+/- 31) with two subjects showing an increase (p = 0.0035). BIS Rinf values decreased by 17.5 Ohms (+/- 9.1) with no subjects showing an increase (p < 0.0001).

**Conclusions:**

Of all the variables, BIA resistance and BIS Rinf measurements appear to be the best detectors of acute changes in hydration with UC failing to detect a change in 31% of the subjects and USG failing in 19% of the subjects. Urinary indices of hydration may not detect an actual loss of fluid after acute dehydration.

**Acknowledgments**

J. Moon and M. Aivazelis are employees of ImpediMed, Inc., the manufacturer of the BIA and BIS devices used in the collection of the bioimpedance data. This study was funded by MusclePharm, Corp.

## P50 Energy drink ingestion effects on barbell velocity and reaction time in women

### Michael Lane, Ryan Bean, Lee Doernte, Katherine Grasberger, John Isaacs

#### Eastern Kentucky University, Richmond, KY, USA

##### **Correspondence:** Michael Lane (Michael.Lane@eku.edu)

**Background**

There is very little research on acute barbell lifting performance alterations due to supplementation in women. Athletic female populations utilizing velocity tracking apparatus to determine performance must be performed to analyze the effects of energy drinks.

**Purpose**

Investigate the effects of an energy drink on barbell lifting, and reaction time in women.

**Methods**

17 college-aged female subjects completed this study (1.64±.08 m, 61.3±6.8 kg, 21.6±1.7y/o, mean±std dev). The initial visit involved consent documentation, health history information, and anthropometrics. Body composition was established using plethysmography (body fat percentage 22.5±6.9%). Subjects were familiarized with the lab’s computerized reaction time test (RTT) (PsychoPy-Python V1.82) and tested for their one repetition maximum (1RM) on back squats (73.2±21.8 kg) and bench press (43.0±13.4 kg). After initial testing, subjects scheduled their next testing session 5-9 days later. Utilizing a crossover double blind study, subjects were given either a supplement, supplement without carbohydrates, or a placebo. Twenty minutes after ingestion subjects took the RTT followed by a warm up and three vertical jumps. For the squat and bench press subjects performed the same protocol of 30, 40, 50, 60, 70, 80, and 90% of their 1RM for up to 3 repetitions at each load. Afterwards subjects performed 15 repetitions on a cadence with 70% of their system max for the movement. Afterwards they performed another RTT. Each visit was 5-9 days from the other trials. Individual lifting performance was normalized and RTT change scores were established. Data was analyzed utilizing two way RMANOVAs with post hoc Bonferroni adjustment for significant differences between treatments.

**Results**

Reaction time test correct answer rate was 89.6±12.19% at a rate of .55±.10 sec. Squat mean velocity performance across the different percentages was: .96±.14, .90±.12, .85±.11, .77±.10, .69±.10, .61±.09, .49±.09 m/s respectively. Bench press mean velocity performance across the different percentages was: .79±.16, .79±.15, .76±.13, .71±.12, .63±.11, .50±.10, .38±.11 m/s respectively. There were no significant differences in any variable for the energy drink conditions compared to the placebo (p > .05).

**Conclusion**

Energy drink consumption does not have an effect on maximal velocity output in squat and bench press performance nor in a fatiguing protocol.

## P51 Energy drink ingestion effects on barbell velocity, reaction time, and mood in men

### Michael Lane, Ryan Bean, Lee Doernte, Katherine Grasberger, John Isaacs

#### Eastern Kentucky University, Richmond, KY, USA

##### **Correspondence:** Michael Lane (Michael.Lane@eku.edu)

**Background**

Use of caffeine and energy drinks might be able to mask fatigue when otherwise an athlete would underperform. Barbell velocity is used as a marker of athlete performance in the collegiate and professional level. Research must be performed into the effects of energy drinks on lifting performance.

Purpose: Investigate the effects of an energy drink on velocity, reaction time, and ratings of perceived exertion.

**Methods**

15 college-aged male subjects participated in this study (1.79±.04 m, 80.3±5.8 kg, 22.7±2.8y/o, mean ± standard deviation). Subjects arrived and filled out participation documents. Body composition was established using air displacement (body fat percentage 14.1±4.9). Subjects were then familiarized with the lab’s computerized reaction time test (RTT) (PsychoPy-Python V1.82). Subjects were tested for their one repetition maximum (1RM) on barbell back squats (129.5±27.5 kg) and bench press (99.7±25.5 kg). Following baseline testing, subjects scheduled their next testing session conducted 5-9 days later. Utilizing a crossover double blind study, subjects were given either a placebo, supplement, or supplement without carbohydrate. After waiting twenty minutes they performed the RTT and visual analog scale (VAS) followed by a standardized warm up and three vertical jumps. Subjects then squatted and performed up to three maximal velocity repetitions with 30, 40, 50, 60, 70, 80, and 90% of their 1RM. Afterwards subjects performed 15 repetitions on a cadence with 70% of their system max. Subjects performed the same protocol on the bench press. Finally, subjects performed the RTT and VAS again. Visits were spaced 5-9 days apart for each trial. Data was normalized for individual lifting performance and change scores were established for the RTT and VAS. Data was analyzed utilizing SPSS with ANOVAs for significant differences between treatments.

**Results**

Mean velocity performance for each percentage on the squat was: 1.11±.19, 1.04±.15, .95±.17, .85±.13, .74±.12, .63±.10, .52±.12 m/s respectively. Mean velocity performance for each percentage on the bench press was: 1.06±.17, .96±.16, .84±.13, .73±.12, .60±.13, .48±.11, .34±.12 m/s respectively. Overall there were no significant differences in performance across any of the percentages, other than the 30% squat. Subjects saw a significant decrease in their anxiety ratings during the supplement trials (p < .05).

**Conclusions**

Energy drinks potentially have a small effect on squat velocity performance for a few points along the 1RM spectrum, but do not significantly affect the entire range.

## P52 The relationship between the ACTN3 genotype and measures of stress, exercise performance and body composition

### Tobin Silver^1^, Jaime Tartar^2^, Sarah Knafo^1^, Anya Ellerbroek^1^, Tobin Silver^1^, Leonel Vargas^1^, Corey Peacock^1^, Jose Antonio^1^

#### ^1^Department of Health and Human Performance, Nova Southeastern University, 3301 College Avenue, Davie FL USA 33314; ^2^Department of Psychology and Neuroscience, Nova Southeastern University, 3301 College Avenue, Davie FL USA 33314

##### **Correspondence:** Jose Antonio (ja839@nova.edu)

**Background**

Alpha-Actinin-3 is a Z-disc protein expressed in fast-twitch or Type II skeletal muscle fibers. A polymorphism in the ACTN3 gene (R577X) results in the XX genotype (i.e., lack of alpha-actinin-3). The purpose of this study was to assess the relationship between the expression of the ACTN3 gene (RR homozygous or RX heterozygous) and measures of stress, bench press strength and endurance, and body composition.

**Methods**

One-hundred and ten male and female athletes volunteered for this study (mean±SD: age 30.2±9.2 years; height 171.3±9.6 cm; % body fat 21.6±7.0; male n=55, female n=55). Body composition was assessed via a DEXA scan. Strength and muscle endurance was determined via a 1-RM for the bench press and repetitions to failure on the bench press at 60% of the 1-RM weight. Saliva was collected before and after the exercise test to determine salivary cortisol concentration as well as for ACTN3 genotyping. Genomic DNA extraction was performed using the QIAamp DNA Investigator kit, following the manufacturer instructions (QIAGEN, Valencia, CA). After isolation, amplification was conducted by polymerase chain reaction (PCR) using allele specific fluorescent primers.

**Results**

There were no significant differences in body composition between XX and R/- (carriers of ACTN3) for body weight, lean body mass, fat mass or percentage body fat. However, R/- demonstrated significantly greater bone mineral content and density. Self-reported stress (pre- and post-exercise testing) showed no differences between XX and R/-. Baseline cortisol was significantly greater in the R/- group; however, no differences were found post-exercise testing. Furthermore, there were no significant differences between the XX and R/- groups for 1-RM bench press strength or repetitions to failure.

**Conclusion**

In exercise-trained individuals, the presence of the ACTN3 genotype did not confer an advantage with muscular strength/endurance, lean body mass or fat mass. However, carriers of the gene (R/-) demonstrated greater bone mineral content and density. Furthermore, the stress response (i.e., self-reported and salivary cortisol) was not different between groups post-exercise.

**Keywords**: ACTN3, DEXA, Body Composition, Cortisol, Strength

## P53 The effect of probiotic supplementation on body composition, muscle thickness, and athletic performance in Division I collegiate athletes

### Jeremy R. Townsend^1^, Jeremy C. Toohey^1^, Sean B. Johnson^1^, Chelsea C. Crimi^1^, Kathryn L. Stowers^1^, William D. Bender^1^, William C. Vantrease^1^, Ann M. Toy^1^, Matthew D. Ruiz^1^, Trisha VanDusseldorp^2^, Yuri Feito^2^, Gerald T. Mangine^2^

#### ^1^Exercise and Nutrition Science, Lipscomb University, Nashville, USA; ^2^Exercise Science and Sport Management, Kennesaw State University, Kennesaw, GA, USA

##### **Correspondence:** Jeremy R. Townsend (jrtownsend@lipscomb.edu)

**Background**

While the use of probiotics for improved immune function and gastrointestinal health has increased in popularity, little is known regarding the effect of supplementation on athletic adaptations and performance. Recent evidence suggests that probiotic supplementation may improve short-term recovery following an acute bout of resistance exercise, which may augment adaptations. Thus, the purpose of this investigation was to determine the effects of probiotic supplementation during offseason training in collegiate athletes.

**Methods**

Twenty-three Division I female athletes (19.6±1.0y, 67.5±7.4 kg, 170.6±6.8 cm) from the university volleyball (n=10) and soccer (n=13) teams participated in this study and were randomized into either a probiotic (DE111; n=11) or placebo (PL; n=12) group. Athletes completed the same 10-week resistance training program during the offseason, which consisted of 3-4 workouts per week of upper and lower-body exercises and sport-specific training. Athletes consumed DE111 (DE111®; 5 billion CFU/day) or PL supplement in conjunction with a recovery drink (45 g CHO, 20 g PRO, 2 g FAT) immediately following resistance and sport-specific training for the entire 10-week program. On weekend or non-training days, athletes consumed the supplement with a meal. Pre and post-training, all athletes underwent one-repetition maximum (1RM) strength testing (squat, deadlift, bench press), performance testing (vertical jump, pro-agility) and isometric mid-thigh pull testing (IMTP). Three compartment body composition estimation (BF%) was completed via BOD POD and BIA analysis, as well as muscle thickness (MT) measurement of the rectus femoris (RF) and vastus lateralis (VL) via ultrasonography. Separate repeated measures analyses of variance were used to analyze all data. Additionally, magnitude based inferences were implemented to provide qualitative interpretation.

**Results**

DE111 produced significantly greater (p=0.015) improvements in BF% (-2.05±1.38%) compared to PL (-0.2±1.6%), with no other group x time interactions observed. Significant improvements were observed for both groups in squat 1RM (p<0.001), deadlift 1RM (p<0.001), bench press 1RM (p<0.001), vertical jump (p<0.001), BF% (p=0.001), and RF MT (p=0.015). No significant main effects were observed for any other variable. Furthermore, DE111 supplementation was “very likely beneficial” for BF% and “possibly beneficial” for deadlift 1RM (+12.0±6.6 kg) compared to PL (+7.8±7.4 kg).

**Conclusions**

These data suggest that probiotic consumption in conjunction with adequate post-workout nutrition may improve body composition and indices of athletic performance in female Division I soccer and volleyball players following offseason training. Future research is needed to elucidate potential mechanisms responsible for these findings.

**Acknowledgements**

This study was supported by Deerland Enzymes Inc.

## P54 The effects of whey protein isolate vs. a reduced volume of a proprietary processed whey protein isolate supplementation in conjunction with resistance training on body composition in resistance trained males

### Bill I. Campbell, Shiva Best, Danielle Aguilar, Andres Vargas, Amey Corson, Ross Perry, Karina Noboa, Paola Fink-Irizarry, Andres Toledo, Barbara Sanchez, Danielle Drywa, Ashley Adams, Natalie Concepcion, David Gaviria

#### University of South Florida, Performance & Physique Enhancement Laboratory, Tampa, FL, USA

##### **Correspondence:** Bill I. Campbell (bcampbell@usf.edu)

**Background**

Recently, a novel whey protein isolate (WPI) processing technique has been introduced, which may improve absorption, digestibility, and ultimately training adaptations. Utilizing this WPI processing technology, the purpose of this investigation was to determine the effects of two different types of whey protein dietary supplements (standard whey protein isolate [Standard WPI] vs. a reduced volume of a proprietary processed whey protein isolate [Novel WPI]) on body composition in conjunction with an 8-week resistance-training program in resistance-trained males.

**Methods**

32 resistance-trained males (22.2±4.3 years; 177.3±7.8 cm; 77.6±12.6 kg) participated in this randomized, double-blinded investigation. Participants were matched according to fat-free mass (FFM) and randomized to the Standard WPI (n=18) or the Novel WPI (n=14). The Standard WPI group was provided with 27 g of WPI per serving and the Novel WPI group was given a reduced volume of WPI (20 g of uniquely processed WPI+7 g maltodextrin to match the volume of the Standard WPI serving size) immediately after each resistance training session (4x/week). At baseline and following 8-weeks of training, participants were assessed for body composition (FFM, dry lean mass [DLM], fat mass [FM], and bodyfat percentage [BF%]). The resistance-training program consisted of two lower-body and two upper-body workouts/week for 8 weeks. Data were analyzed via a 2-factor [2x2] between-subjects repeated measures ANOVA and pre to post changes within each group were analyzed by a paired-samples t-test.

**Results**

No differences existed between the two groups for body composition measures at baseline. The repeated measures ANOVA revealed a main effect for time for FFM (p<0.001) and DLM (p=0.05), but no group x time interactions. Specifically, FFM increased from 68.8±9.3 kg to 70.0±9.4 kg and from 67.1±9.0 kg to 67.8±9.7 kg; DLM increased from 19.6±3.7 kg to 20.2±3.5 kg and from 19.3±3.6 kg to 20.1±5.2 kg in the Standard WPI and Novel WPI groups, respectively. The paired samples t-test revealed a significant increase in FFM over time in the Standard WPI group (p=0.001) and a trend for significance in the Novel WPI group (p=0.082). However, when body water was accounted for (DLM), neither group significantly increased DLM over time (Standard WPI: p=0.164; Novel WPI: p=0.185). There were no main effects for time nor a group x time interaction for FM and BF% (p>0.05).

**Conclusions**

In resistance-trained males, using a reduced amount (25% less WPI) of novel processed WPI as a post-workout protein supplement elicits changes in body composition similar to using a higher-protein dosed, standard WPI supplement.

**Acknowledgements**

This study was supported by Plasma Nutrition.

## P55 The effects of whey protein isolate vs. a reduced volume of a proprietary processed whey protein isolate supplementation in conjunction with resistance training on maximal strength in resistance trained males

### Shiva Best, Bill I. Campbell, Chris Gai, Kevin Hartke, Dante Xing, Carl Fox, Seth Donelson, Brad Simon, Matthew R. Wynn, Maria De La Torre, Josh Rubio, Christine Lodato, Delaney Troop

#### University of South Florida, Performance & Physique Enhancement Laboratory, Tampa, FL, USA

##### **Correspondence:** Bill I. Campbell (bcampbell@usf.edu)

**Background**

Recently, a novel whey protein isolate (WPI) processing technique has been introduced to the market, which may improve absorption, digestibility, and ultimately training adaptations. Utilizing this WPI processing technology, the purpose of this investigation was to determine the effects of two different types of whey protein dietary supplements (standard whey protein isolate [Standard WPI] vs. a reduced volume of a proprietary processed whey protein isolate [Novel WPI]) on maximal strength in conjunction with an 8-week resistance-training program in resistance trained males.

**Methods**

32 resistance-trained males (22.2±4.3 years; 177.3±7.8 cm; 77.6±12.6 kg) participated in this randomized, double-blinded investigation. Participants were matched according to FFM and randomized to the Standard WPI (n=18) or the Novel WPI (n=14). The Standard WPI group was provided with 27 g of WPI per serving and the Novel WPI group was given a reduced volume of WPI (20 g of uniquely processed WPI+7 g maltodextrin to match the volume of the Standard WPI serving size). Both protein supplements were taken immediately after each training session (4x/week). Both groups performed the same training program, and maintained a protein intake of 1.5-2.5 g/kg/d to facilitate recovery from and adaptation to training. At baseline and following 8-week training program, participants were assessed for maximal strength on the back squat, bench press, and deadlift. The program consisted of two lower-body and two upper-body workouts/week for an 8-week period. Data were analyzed via a 2-factor [2x2] between-subjects repeated measures ANOVA using SPSS v22.0. The criterion for significance was set at p≤0.05.

**Results**

No differences existed between the two groups for strength measures at baseline. The repeated measures ANOVA revealed a main effect for time for the back squat (p<0.001), bench press (p<0.001), and deadlift (p<0.001) exercises, but no group x time interactions were observed for absolute or relative strength between groups. Specifically, back squat increased from 131.2±25.5 kg to 144.8±25.1 kg (improvement of 10.4%) and from 131.6±37.6 kg to 145.5±35.4 kg (improvement of 10.6%); bench press increased from 100.3±19.0 kg to 108.0±19.5 kg (improvement of 7.7%) and from 96.0±19.9 kg to 100.9±20.2 kg (improvement of 5.1%); deadlift increased from 151.0±33.3 kg to 162.0±31.1 kg (improvement of 7.3%) and from 149.6±31.9 kg to 158.7±35.3 kg (improvement of 6.1%) in the Standard WPI and Novel WPI groups, respectively.

**Conclusions**

In resistance-trained males, using a reduced amount (25% less WPI) of novel processed WPI as a post-workout protein supplement elicits the same increases in strength as a higher-protein dosed, standard WPI supplement.

**Acknowledgements**

This study was supported by Plasma Nutrition.

## P56 The effects of shred sport on metabolic rate

### Ross Perry, Bill Campbell, John Horsley, Danielle Aguilar, Taylor Shimshock, Andres Vargas, Carl Fox, Kelly Northrop, Seth Donelson

#### University of South Florida, Performance & Physique Enhancement Laboratory, Tampa, FL, USA

##### **Correspondence:** Bill Campbell (bcampbell@usf.edu)

**Background**

The purpose of this study was to investigate the effects of a commercially available thermogenic supplement on resting metabolic rate (RMR) and hemodynamic variables in healthy males by using a randomized, double-blind, placebo-controlled crossover design.

**Methods**

Ten males (26.5±6.4 years, 177.6±7.2 cm, 80.5±10.8 kg) participated in the investigation. Each participant underwent two different testing sessions within a 7-day period. After an overnight fast, each session began with the collection of heart rate (HR) and blood pressure (BP) assessments, as well as two baseline RMR measurements. Following this, each participant ingested either a thermogenic supplement (Shred Sport™) or a placebo and the RMR, HR, and BP assessments were repeated at 60, 120, and 180 minutes post-ingestion. Data were analyzed via a 2-factor [2x4] within-subjects repeated measures ANOVA using SPSS version 22.0.

**Results**

Repeated measures ANOVA revealed a significant effect for time relative to the raw RMR data. Post-hoc analyses revealed that the dietary supplement treatment demonstrated significant elevations in RMR at 60-minutes, 120-minutes, and 180-minutes post-ingestion (p ≤ 0.05). There were no significant elevations at any time point in the placebo treatment. Table 12 demonstrates the raw data and the percentage increases in RMR for each time point for both supplement treatments. HR significantly decreased at 60-minutes (from 60.3 ± 7.3 to 55.2 ± 5.0) and 180-minutes (from 60.3 ± 7.3 to 56 ± 4.7 beats/minute) in the thermogenic treatment and did not change at any time in the placebo treatment. SBP did not change significantly at any time point in either treatment. DBP increased significantly at all time points in the thermogenic treatment and increased significantly at 180-minutes post-ingestion in the placebo treatment, but all measures stayed within normal clinical values (60-80 mm Hg).

**Conclusions**

The dietary supplement treatment (Shred Sport™) experienced significant elevations in RMR— an effect that was not observed with placebo treatment. Taken on a daily basis, Shred Sport™ supplementation may increase overall energy expenditure possibly leading to reductions in fat mass over time.

**Table 12 (abstract P56).** RMR (mean ± SD kcals/day) and (% increase in RMR as compared to baseline values) for each supplement group

	Baseline	60-minute	120-minute	180-minute
Shred Sport^TM^	1,859 ± 266	2,027 ± 288 (9.0%)*	2,072 ± 292 (11.5%)*	2,040 ± 271 (9.7%)*
Placebo	1,963 ± 358	2,014 ± 422 (2.6%)	1,991 ± 300 (1.4%)	2,010 ± 358 (2.4%)

* - Post-hoc statistical difference compared to baseline values (p ≤ 0.05)

## P57 Tiered vs. Traditional Daily Undulating Periodization for improving body composition in trained males

### Andres Vargas, Bill Campbell, Danielle Aguilar, Paola Fink-Irizarry, Ross Perry, Ari Zucker, Ashlynn Paskert, Emily Rzonca, Kelly Northrop, Andres D. Toledo, Jordan Reyes, Brad Simon, Kate Germain, Josh Rubio, Rena Jo Griggs, Rachael Jimenez, Karina Noboa, Kyshia Chernesky

#### University of South Florida, Performance & Physique Enhancement Laboratory, Tampa, FL, USA

##### **Correspondence:** Bill Campbell (bcampbell@usf.edu)

**Background**

Daily undulating periodization (DUP) represents an increasingly popular trend in exercise science with which a traditional model has been established. The purpose of this study was to compare the effects of traditional and tiered DUP models as they relate to body composition changes in trained males.

**Methods**

27 resistance-trained males (22±4.5 years; 80±11 kg) completed an 8-week resistance-training program and were randomly assigned to one of two groups: Traditional Daily Undulating Periodization (DUP; n=12) or Tiered Daily Undulating Periodization (TDUP; n=15). All subjects were required to squat, bench press, and deadlift 1.25x, 1x, and 1.5x their bodyweights, respectively. DVs included fat free mass (FFM), fat mass (FM), and body fat percentage (BF%) and were assessed at baseline and after 8-weeks of training. The DUP group performed a hypertrophy-workout on Monday, a power-workout on Wednesday, and a strength-workout on Friday. The last set of all 3 powerlift exercises was taken to failure during the strength workout such that the performance of this set dictated the working weight for each lift the following week. In contrast, the TDUP group emphasized one powerlift for each workout and completed their last set of their emphasized lift to failure. Data was analyzed via a 2x2 mixed factorial ANOVA with the alpha criterion for significance set at 0.05.

**Results**

There were no significant differences in total volume or intensity between groups. There was a main effect for time (p=0.008) for FFM (DUP pre=73.3±8.3 kg, DUP post=74.9±9.4 kg; TDUP pre=68.7±8.2 kg, TDUP post=69.6±8.6 kg). This was representative of a 1.2 kg average increase in FFM over 8 weeks. No significant effects for time were observed for FM (p=0.812; DUP pre= 9.1±4.3 kg, DUP post=9.6±4.8 kg; TDUP pre=9.5±4.3 kg, TDUP post=9.3±4.1 kg) or BF% (p=0.241; DUP pre=10.7±3.6%, DUP post=10.9±3.8%; TDUP pre=11.9±4.3%, TDUP post=11.5±3.8%) after 8 weeks. There were no interaction effects between the DUP and TDUP for any of the body composition variables assessed.

**Conclusions**

8-weeks of tiered DUP leads to comparable gains in FFM when compared to a traditional DUP program in trained males. This may be attributed to the fact that both groups performed similar volumes of work throughout the study. Specifically, DUP increased FFM by 1.6 kg, while TDUP increased FFM by 0.9 kg. Non-significant changes were observed for fat mass and BF% such that the DUP increased FM and BF% while the TDUP saw slight decreases in both variables.

**Acknowledgements**

This investigation was supported by Dymatize Athletic Nutrition Institute (DANI).

## P58 The acute effects of weighted vest running on substrate oxidation and energy expenditure

### T. M. Purdom^1,2^, L. Kravitz^2^, C. Mermier^2^, T. Moriarty^2^, N. Cole^2^, K. Johnson^2^

#### ^1^Department of Health, Athletic Training, Recreation, and Kinesiology, Longwood University, Farmville, VA 23909; ^2^Department of Health, Exercise, and Sports Sciences, University of New Mexico, Albuquerque, NM 87131

**Background**

Lipids are the substrate largely responsible for energy supply during submaximal exercise [1]. However, the contribution of substrates (FA and CHO) oxidized for energy permutate as exercise intensity increases leading to the crossover point where CHO is the dominant substrate oxidized [2]. Weighted vest running (WVR) has been shown to acutely modify running kinematics, which can impact exercise intensity [3]. Therefore, the purpose was to assess the influence of WVR with an additional 5%BM and 10%BM on caloric expenditure (CE) and FAox.

**Methods**

Seventeen recreationally trained runners (9 men and 8 women) performed four separate graded exercise tests (GXT) separated by 24 hrs each. After height and weight were measured, the sum of three skinfold sites was used to estimate percent body fat prior to the first GXT. The first GXT established the workloads at pre-specified exercise intensities (60,65,70,75,80% VO_2max_). The following three GXTs tested WVR with a control (no vest), 5% body mass (BM) vest, and 10%BM vest using 3-minute incrementally increasing steady-state stages. Indirect calorimetry was used to measure both FAox (g/min) and CE (kcal/min).

**Results**

The ANOVA/ANCOVA analysis revealed that WVR significantly increased CE (*p* < 0.05) and reduced FAox (*p* < 0.05). When sex, fat free mass (FFM), and fat mass (FM) were included as covariates, FFM (kg) was found to have a significant influence (*p* < 0.001) on CE. Fat mass was found to have the strongest influence on FAox (*p* = 0.07) as compared to FFM and sex. Caloric expenditure significantly increased in the 10%BM condition at all exercise intensities compared with the control and 5%BM (except 60% VO_2max_), while FAox significantly decreased in the 10%BM WV (70% and 75% VO_2max_ conditions only).

**Conclusions**

Our results agree with previous research that 60% VO_2max_ elicits maximal FAox [1]. However, our results show that recreationally conditioned runners training with weighted vests at 5%BM and 10%BM will increase CE while maintaining fat oxidation. The 10%BM had the most meaningful increase in CE. Furthermore, our results confirm that symetrical placement of mass has little effect on exercise intensity while running. These findings are relevant for running-based exercise prescriptions to increase CE, but maintain fat specific substrate oxidation. Trained runners maintaining a higher FM (within reason) could positively impact FAox. Conversely, FFM precipitates total energy expenditure, therefore, increasing FFM can positively increase CE during running activities. Future investigations should consider body composition of importance when exploring substrate oxidation.

**References**

1) Achten, J, Jeukendrup, AE. Optimizing fat oxidation through exercise and diet. *Nutrition. 2004;*20:716-727.

2) Brooks, G, Mercier, J. Balance of carbohydrate and lipid utilization during exercise: the “crossover” concept. *Am Phys Soc.* 1994;76(6):2253-2261.

## P59 Six grams of fish oil supplementation improves recovery of indirect markers of muscle damage following eccentric exercise

### Matthew Lee^1^, Trisha A. VanDusseldorp^1^, Kurt A. Escobar^2^, Kelly E. Johnson^2^, Matthew Stratton^2^, Terence Moriarty^2^, James J. McCormick^2^, Gerald T. Mangine^1^, Alyssa Holmes^1^, Nathan Cole^1^, Chad M. Kerksick^3^, Christine Mermier^2^

#### ^1^Department of Exercise Science and Sport Management, Kennesaw State University, Kennesaw, GA, 3011, USA; ^2^Department of Health, Exercise and Sports Sciences, University of New Mexico, Albuquerque, NM, 87131, USA; ^3^Department of Exercise Science, Lindenwood University, St. Charles, MO, 63301, USA

##### **Correspondence:** Trisha A. VanDusseldorp (tvanduss@kennesaw.edu)

**Background**

To examine the effect of fish oil (FO) supplementation on recovery of indirect markers of muscle damage following eccentric exercise.

**Methods and Materials**

Thirty-two, college-aged, resistance trained males (n = 16) and females (n = 16) supplemented with 2.0, 4.0, 6.0 g·d^-1^ FO or placebo (PL) for 7 weeks. Participants then completed a muscle damaging resistance exercise protocol (10 sets of 8 four-second eccentric squats at 70% one-repetition maximum and 5 sets of 20 split-squat jumps). All subjects had blood drawn 8 times during the study: baseline (BL), pre-exercise (PRE), immediately post-exercise (IPE), and 2, 4, 24, 48 and 72 hours (HR) after exercise. Blood samples were collected for serum acquisition. The blood aliquots were centrifuged at 1650 g for 10 minutes and stored at -80 °C until time of analysis. Commercially available kits and a spectrophotometer (Spectramax M3, Molecular Devices) were used for the assessment of creatine kinase (CK; Pointe Scientific) and lactate dehydrogenase (LDH; Pars Azmoon).

**Results**

Group x time interactions were observed for CK (F = 2.63, *p* = 0.018, n2 = 0.22) and LDH (F = 4.00, *p* < 0.001, n2 = 0.30). Significant (*p* < 0.05) main effects for time were observed for all dosage groups in relation to BL and PRE concentrations (CK & LDH). Compared to PL (CK: 1804.9 ± 2034.8 IU/L; LDH: 410.0 ± 200.3 IU/L), CK tended (*p* = 0.055) to be lower at 72-HR for 6 g·d^-1^ (114.3 ± 21.1 IU/L) while LDH tended to be lower for 6 g·d^-1^ at 24-HR (194.2 ± 49.2 IU/L) and 48-HR (198.0 ± 55.0 IU/L) before significantly (*p* = 0.005) lower concentrations were observed at 72-HR (130.9 ± 28.3 IU/L). At 24-HR, lower (*p* = 0.020) CK concentrations were observed in 6 g·d^-1^ (544.5 ± 150.8 IU/L) compared to 2 g·d^-1^ (3020.6 ± 1753.4 IU/L). At 48-HR, CK concentrations tended (*p* = 0.076) to be lower in 6 g·d^-1^ (261.1 ± 103.5 IU/L) compared to 4 g·d^-1^ (2188.3 ± 2110.3 IU/L). At 72-HR, lower (*p* = 0.005) LDH concentrations were observed in 6 g·d^-1^ compared to 2 g·d^-1^ (412.1 ± 135.0 IU/L).

**Conclusion**

Ingestion of 6 g·d^-1^ FO improves recovery of indirect markers of muscle damage following eccentric exercise.

**Acknowledgments**

Supported by the International Society of Sports Nutrition and MusclePharm Grant.

## **P60 Effects of tart cherry extract on regenerating** C2C12 **skeletal myotube cultures and murine macrophages**

### Kevin S. O’Fallon^1^, Benjamin B. Johnson^1^, Andrew Lee^1^, Margery G.H. Pelletier^2^, Klaudia Szymczak^2^, Anna M. Barbeau^2^, Peter Gaines^2^

#### ^1^Combat Feeding Directorate, Integrative Physiology Laboratory, U.S. Army Natick Soldier RDEC, Natick, MA, USA; ^2^Department of Biological Sciences, Biomedical Engineering and Biotechnology Program, University of Massachusetts Lowell, Lowell, MA, USA

**Background**

The inflammatory response to exercise induced muscle damage (EIMD) involves sequential activation of pro-(M1) and anti-inflammatory(M2) macrophage phenotypes, which corresponds to the destructive and regenerative phases of muscle remodeling/repair. Daily consumption of tart cherry juice before and after EIMD has been shown to accelerate strength recovery in humans; a purported benefit attributed to antioxidant/anti-inflammatory phytonutrients in tart cherries. However, mechanisms of such compounds to influence damaged skeletal myofibers, and the macrophages that aid in their regeneration are poorly understood. The purpose of this study was to investigate the effects of tart cherry extract (TCE) *in vitro* on regenerating C2C12 skeletal myotube cultures, and *ex vivo*-cultured murine macrophages.

**Materials & Methods**

C2C12 cultures were pretreated with 30 μg/ml TCE for 24 h prior to experiments. A FlexCell™ FX5000 was used to impart mechanical strain on cultures for 0, 6, or 18 h. Cultures were then assayed at all time points for Hepatocyte Growth Factor (HGF) and Monocyte Chemoattractant Protein-1 (MCP-1) by ELISA, Myosin Heavy Chain (MHC) by Western blot, and satellite cell (SC) activation by EdU Imaging Kit. Bone marrow-derived myeloid cells were used as an *ex-vivo* model of macrophage differentiation. Macrophage populations included three subtypes: M0(unstimulated), M1(activated by IFNγ&LPS), and M2(activated by IL-4). Phenotypes were induced and confirmed by immunolabeling cell surface/protein complex markers [M0(Mac-1,F4/80), M1(CD11c,iNOS), M2(MGL1/2,CD206)], Imaging Flow Cytometry, and Western blot, in presence/absence of TCE.

**Results**

TCE pretreatment significantly (p<0.05) altered C2C12 cell responses to mechanical strain in several ways. First, MCP-1 secretion increased by 48%±11%, and HGF release increased by 10%±0.39% among TCE-treated/injured cultures after 18 h strain, versus untreated/injured cultures. Second, MHC expression increased by 131%±43% after 18 h strain, over untreated/injured cultures. Third, and unexpectedly, TCE decreased SC activation by 80%±8% after 6 h, and 90%±34% after 18 h of strain, versus untreated/injured cultures. Preliminary findings among M0, M1, and M2 macrophages suggested similar expression levels of cell surface markers Mac-1 and F4/80 across phenotypes. Upregulation of iNOS in M1 and Arginase-1 in M2 macrophages, over unstimulated controls was also observed, demonstrating the reliability of the *ex vivo* model. Preconditioning M1 macrophages for 48 h in 25 μg/ml TCE decreased iNOS expression, relative to untreated controls, suggesting TCE may regulate the pro-inflammatory enzymatic system (iNOS) utilized by M1 macrophages.

**Conclusion**

This novel research demonstrates effects of TCE on specific mechanistic functions critical to muscle regeneration in both muscle and immune cells.

**Acknowledgements**

Funding Source: Combat Feeding Research and Engineering Program

## P61 Native whey supplementation, recovery and training gains

### Vincent Martin^1^, Sebastian Garcia-Vicencio^1^, Céline Gryson^1^, Enzo Pipnnier^1^, Jacqueline Brasy^2^, Pascale Le Ruyet^2^, Marion Bucas^3^, Victoire Visseaux^4^, Yann Connan^4^, Florence Montel^5^, Clément Lahaye^5^_,_ Yves Boirie^6^, Sébastien Ratel^1^

#### ^1^Université Clermont Auvergne, AME2P, CRNH Auvergne, F-63000, Clermont-Ferrand, France; ^2^Lactalis R&D, F-35500, Retiers, France; ^3^Lactalis Ingredients, F- 35230, Bourgbarré, France; ^4^Lactalis Ingredients, Buffalo, NY, 14220, USA; ^5^CHU Clermont-Ferrand, Service Nutrition Clinique, F-63000, Clermont-Ferrand, France; ^6^Université Clermont Auvergne, INRA, UNH, Unité de Nutrition Humaine, CRNH Auvergne, F-63000, Clermont-Ferrand, France

##### **Correspondence:** Vincent Martin

**Background**

Electrostimulation training (ES) can increase muscle power, but this aggressive modality of training may also increase muscle fatigue and possibly delay recovery (1). It has been demonstrated that Native whey (NW) supplementation, may reduce neuromuscular fatigue induced by strength training programs (2, 3).

The aim of this study was therefore to evaluate the impact of NW supplementation and a training program consisting in ES and voluntary training, on recovery and training gains.

**Material and methods**

Forty-two moderately active men (21.5±3.2 years) were involved in this pilot, double-blinded trial and followed a 12-week training program; 6 weeks ES (3/week) and 6 weeks ES combined with plyometric and sprint training sessions (2-3/week). They were randomly allocated into 3 groups supplemented 5d/week with one of three isocaloric beverages; 30 g of carbohydrates (Control), or 15 g of carbohydrates and 15 g of protein from NW or from standard whey protein isolate (WPI).

Recovery of maximal concentric power (Pmax) was measured before, immediately after, and 30 min, 60 min, 24 h, 48 h after three ES sessions; the first (at week1 (W1)), the fourth (W2) and last (W12). Muscle performance was evaluated before (W0), after 6 (W6) and 12 weeks of training (W12); maximal voluntary contraction force (CMV), evoked twitch amplitude (Pt), anatomical cross-sectional area (CSA) and maximal voluntary activation level (VAL) of the knee extensors were measured.

**Results**

Pmax recovery was initiated at 30 min in NW, 24 h in WPI and 48 h in Control (P<0.01). Training gains also differed between groups, with CMV increase between W0 and W12 by +11.8% in NW (P<0.001), +7.1% in WPI (P<0.05), and no change in Control. However, between W6 and W12, only NW showed gains in CMV. VAL remained unchanged in NW throughout the follow-up, while it decreased at W12 in WPI (-3.5%, P<0.05), and at W6 and W12 in Control (-3.9%, P<0.05). Pt and CSA improved as a result of training, and did not differ between groups.

**Conclusions**

As suggested by these results, NW, as compared to WPI and Control, initiates power recovery process earlier. The strength gains observed along the entire training period in NW are possibly associated with this early recovery process, and could be explained not only by the muscle hypertrophy observed in all groups, but also by the preserved VAL, that decreased in WPI and Control, suggesting that overtraining occurred in these 2 groups

**Author Disclosure Information**

**V. Martin :** Contracted Research - Including Principle Investigator;

Study funded by Lactalis Ingredients.

**References**

1. Zory RF, Jubeau MM, and Maffiuletti NA : Contractile impairment after quadriceps strength training via electrical stimulation. Journal of strength and conditioning research 2010, 24(2):458-64.

2. Babault N, Deley G, Le Ruyet P, Morgan F, and Allaert FA : Effects of soluble milk protein or casein supplementation on muscle fatigue following resistance training program: a randomized, double-blind, and controlcebo-controlled study. Journal of the International Society of Sports Nutrition 2014, 11-36.

3. Gryson C, Ratel S, Rance M, Penando S, Bonhomme C, Le Ruyet P, Duclos M, Boirie Y, and Walrand S : Four-month course of soluble milk proteins interacts with exercise to improve muscle strength and delay fatigue in elderly participants. Journal of the American Medical Directors Association 2014, 15(12):958e1-9.

